# Ruxolitinib induces apoptosis and pyroptosis of anaplastic thyroid cancer via the transcriptional inhibition of DRP1-mediated mitochondrial fission

**DOI:** 10.1038/s41419-024-06511-1

**Published:** 2024-02-09

**Authors:** Ya-wen Guo, Lei Zhu, Yan-ting Duan, Yi-qun Hu, Le-bao Li, Wei-jiao Fan, Fa-huan Song, Ye-feng Cai, Yun-ye Liu, Guo-wan Zheng, Ming-hua Ge

**Affiliations:** 1 Otolaryngology & Head and Neck Center, Cancer Center, Department of Head and Neck Surgery, Zhejiang Provincial People’s Hospital (Affiliated People’s Hospital), Hangzhou Medical College, Hangzhou, Zhejiang 310014 China; 2 Department of Public Health, Zhejiang University School of Medicine, Hangzhou, Zhejiang 310014 China; 3 Key Laboratory of Endocrine Gland Diseases of Zhejiang Province, Hangzhou, Zhejiang 310014 China; 4 Clinical Research Center for Cancer of Zhejiang Province, 310014 Hangzhou, Zhejiang China; 5 Department of Thyroid Surgery, The Fifth Hospital Affiliated to Wenzhou Medical University, Lishui Central Hospital, Lishui City, Zhejiang 323000 China; 6 School of Information Science and Engineering, Zhejiang Sci-Tech University, Hangzhou, Zhejiang 310018 China; 7 Department of Thyroid Surgery, The First Affiliated Hospital of Wenzhou Medical University, Wenzhou, Zhejiang 325000 China; 8 Second Clinical Medical College, Zhejiang Chinese Medical University, Hangzhou, 310053 Zhejiang China

**Keywords:** Targeted therapies, Head and neck cancer

## Abstract

Anaplastic thyroid carcinoma (ATC) has a 100% disease-specific mortality rate. The JAK1/2-STAT3 pathway presents a promising target for treating hematologic and solid tumors. However, it is unknown whether the JAK1/2-STAT3 pathway is activated in ATC, and the anti-cancer effects and the mechanism of action of its inhibitor, ruxolitinib (Ruxo, a clinical JAK1/2 inhibitor), remain elusive. Our data indicated that the JAK1/2-STAT3 signaling pathway is significantly upregulated in ATC tumor tissues than in normal thyroid and papillary thyroid cancer tissues. Apoptosis and GSDME-pyroptosis were observed in ATC cells following the in vitro and in vivo administration of Ruxo. Mechanistically, Ruxo suppresses the phosphorylation of STAT3, resulting in the repression of DRP1 transactivation and causing mitochondrial fission deficiency. This deficiency is essential for activating caspase 9/3-dependent apoptosis and GSDME-mediated pyroptosis within ATC cells. In conclusion, our findings indicate DRP1 is directly regulated and transactivated by STAT3; this exhibits a novel and crucial aspect of JAK1/2-STAT3 on the regulation of mitochondrial dynamics. In ATC, the transcriptional inhibition of DRP1 by Ruxo hampered mitochondrial division and triggered apoptosis and GSDME-pyroptosis through caspase 9/3-dependent mechanisms. These results provide compelling evidence for the potential therapeutic effectiveness of Ruxo in treating ATC.

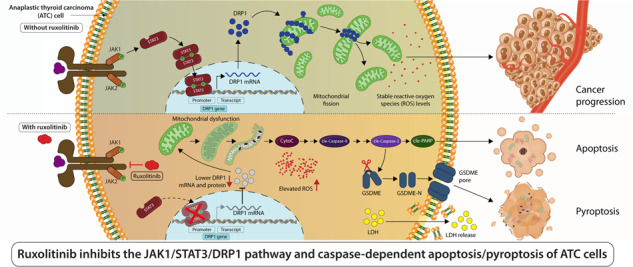

## Introduction

Thyroid cancer is a prevalent form of endocrine malignancy; its incidence is increasing annually worldwide [1], with an age-standardized incidence rate of 10/100,000 in women and 3/100,000 in men in 2020 [2]. Among all types of thyroid cancers, anaplastic thyroid carcinoma (ATC) is the most malignant and has the poorest prognosis. It accounts for approximately 5% of all thyroid cancers; the median survival time of patients is 4−6 months [3], and the disease-specific mortality rate can reach 100%. ATC progresses rapidly and easily invades surrounding tissues, causing local infiltration and cervical lymph node metastasis. Notably, even in the early stages, distant metastasis is a possibility. Patients frequently succumb to asphyxia, a consequence of either localized tumor growth or the dissemination of cancer cells to distant sites [4]. The existing therapeutic approaches for ATC, encompassing surgical resection, chemotherapy, and combination therapy, exhibit limited efficacy in disease management. For instance, the United States Food and Drug Administration (FDA)-approved drugs Trametinib and Dabrafenib, utilized for the treatment of ATC with BRAF^V600E^ mutations, are only effective in a limited subset of patients and may elicit severe adverse reactions. This underscores the urgent need to pinpoint novel targets and strategies for ATC management.

JAK/STAT signaling pathway is widely known in tumor research due to its involvement in tumor cell proliferation, differentiation, survival, epithelial-to-mesenchymal transition, and immune escape [5]. The JAK/STAT pathway involves JAK1/2 and STAT1-6, with JAK1/2-STAT3 receiving the most attention because of its core regulatory role in malignant tumor progression [6], such as in hepatocellular [7], colorectal [8], cervical [9] and bladder cancer [10]. Due to STAT3, a transcription factor still considered a challenging therapeutic target, the strategy of blocking the upstream receptor and non-receptor kinases, such as JAK1/2, has potential applications in the treatment of and research on JAK1/2-activated tumors [11]. Promising clinical and preclinical studies have shown that FDA-approved JAK inhibitors such as ruxolitinib (Ruxo), fedratinib, tofacitinib, and upadacitinib has substantially reduced JAK-STAT3 activation. Furthermore, these inhibitors have showcased promising therapeutic outcomes across various inflammatory and neoplastic disorders [12, 13]. However, except for Ruxo, there is a scarcity of reports regarding using JAK inhibitors in managing solid tumors [14]. Ruxo is a first-generation selective inhibitor of JAK1 and JAK2 in the JAK/STAT3 pathway [12], has gained clinical approval primarily for treating myeloproliferative neoplasms [15], rheumatoid arthritis, inflammatory bowel disease [13], and a variety of immune-driven skin diseases [16]. In addition, Ruxo has significant anti-cancer effects on solid tumors (hepatocellular [17], breast [18], prostate [19], pancreatic cancer [20], and head and neck cancer [21]). However, to date, the effect of Ruxo has not been reported in ATC target therapy.

The induction of tumor cell death remains a focal point in contemporary anti-tumor research strategies. While a multitude of anti-cancer techniques, including radiotherapy, chemotherapy, and targeted therapy, strive to trigger tumor cell apoptosis, a significant portion of tumor cells exhibit resistance to this process and its subsequent treatments. Pyroptosis, initially proposed by Brennan et al. in 2001, represents a form of programmed cell death reliant on inflammatory caspases and the gasdermin (GSDM) family. This mode of cell death has emerged as one of the promising and innovative strategies in anti-tumor therapies [22]. Various stimuli can elicit the fragmentation of GSDM family proteins through distinct pathways, initiating pyroptosis and releasing inflammatory agents, such as LDH, IL-1β, and HMGB1. Among the GSDM family proteins, GSDMD and GSDME stand out as examples that can be cleaved by caspases, thereby inducing pyroptotic effects [23, 24]. Research has indicated that TNF or chemotherapeutic drugs can transform caspase-3-mediated apoptosis into pyroptosis in tumor cells with GSDME upregulation [23]. The cleavage of GSDME relies on the activation of caspase 3, which can occur through either mitochondria-dependent or mitochondria-independent pathways [25]. Various stimuli, originating from within or outside the cell, can trigger the mitochondria-dependent pathway. This activation leads to a reduction in the potential of the mitochondrial membrane, an elevation in its permeability, and the subsequent release of pro-apoptotic factors such as cytochrome C (Cyto C), apoptosis-inducing factor (Apaf-1), and second mitochondria-derived activator of caspases into the cytoplasm [26]. Cyto C, once released from the mitochondria, combines with Apaf-1 to create an apoptosis complex that enlists and triggers pro-caspase 9 [27]; subsequently, pro-caspase 3 initiates the caspase cascade reaction and cleaves α-tubulin, poly ADP-ribose polymerase (PARP), and GSDME in cells, ultimately leading to programmed cell death [28, 29]. In previous investigation of our center (Otolaryngology & Head and Neck Center, Cancer Center, Department of Head and Neck Surgery, Zhejiang Provincial People’s Hospital), we made a significant discovery concerning the expression of GSDME in ATC cells [30]. The findings strongly indicate that triggering mitochondrial pathway-dependent pyroptosis within these cells is potentially an effective therapeutic approach for ATC [30]. Therefore, exploring effective strategies for inducing pyroptosis in ATC cells may facilitate the advancement of potential therapeutic approaches for ATC.

This study successfully verified the activation of the JAK1/2-STAT3 signaling pathway within the tumor tissues of ATC patients, which showed a distinct contrast when compared to papillary thyroid cancer (PTC) and normal thyroid (NT) tissues. Notably, Ruxo emerged as a significant player, demonstrating its ability to effectively suppress JAK1/2-STAT3 activation. Consequently, it exhibited profound inhibitory effects on cell viability and led to the induction of mitochondria-dependent apoptosis and GSDME-pyroptosis in ATC cells. Mechanistically, Ruxo mediated significant repression of DRP1 transactivation, leading to mitochondrial fission deficiency and mitochondrial depolarization. Notably, we observed and confirmed that DRP1 is directly regulated and transactivated by STAT3. In summary, the present study elucidated that the JAK1/2-STAT3 signaling pathway directly regulates mitochondrial dynamics, and Ruxo is a potential therapeutic agent for treating ATC.

## Materials and methods

### Drugs and reagents

Ruxolitinib (Ruxo, Cat: T1829), Mdivi-1 (Cat: T1907), STX-0119 (Cat: T60160), Tween 80 (Cat: T13947) and PEG300 (Cat: T7022) were procured from TargetMol (Shanghai, China) and DAPI (4′,6-Diamidine-2′-phenylindole dihydrochloride, Cat: C1002) was purchased from Beyotime Biotechnology, China. Z-VAD-FMK (Cat: A1902) was purchased from Apex Bio (Houston, TX, USA). The mitochondrial tracker deep red (Mito tracker) (Cat:8778) was purchased from Cell Signaling Technology (MA, USA). The anti-cytochrome C antibody (Cat:556432) was purchased from BD Biosciences (CA, USA). The anti-HSP60 antibody (Cat: 66041-1-Ig) was purchased from Proteintech (Wuhan, China).

### Patient information and specimens

Tumor tissue specimens were procured from a collective of 4 patients diagnosed with ATC and 10 patients with PTC. Additionally, normal thyroid (NT) tissues were collected from 10 patients diagnosed with benign nodular goiter, who underwent surgery at the Zhejiang Provincial People’s Hospital between January 2016 and December 2022. The excised specimens were promptly cryopreserved in liquid nitrogen at −80 °C. In accordance with the criteria set by the World Health Organization, all specimens were reviewed by two pathologists in a retrospective manner to validate the histological diagnosis. Consent forms were duly acquired from all participating individuals as a measure of ethical responsibility. Approval number QT20222394 was granted by the Ethics Committee of Zhejiang Provincial People’s Hospital for this study.

### Cell culture

The human thyroid epithelial cell line Nthy-ori 3-1 (NTHY), PTC cell lines TPC-1, BCPAP, and IHH4 were acquired from Procell Life Science & Technology (Wuhan, China). The human ATC cell lines KHM-5M, C643, CAL-62 and BHT101 were acquired from the National Collection of Authenticated Cell Cultures (Shanghai, China). Deutsche Sammlung von Mikroorganismen und Zellkulturen provided us with another ATC cell line called 8505C. All cells have STR Authentication and were cultured in HyClone RPMI-1640 with 10% fetal bovine serum (Cat: S-FBS-SA-015, SERANA, Germany), 100U/ml penicillin, and 0.1 mg/ml streptomycin. The cells were maintained at 37 °C with 5% CO_2_.

### Cell viability

The viability of cells was estimated using a CCK-8 assay (Cat: A311-01; Vazyme Biotech Co., Ltd., China) according to the instructions provided by the manufacturer. Prior to treatment, the cells were placed in 96-well plates at a density of 5000 cells per well and incubated at 37 °C with 5% CO_2_ for 24 h. The vehicle solvent dimethyl sulfoxide (DMSO) was added at a concentration of 1 µL/ml or lower, and different concentrations of Ruxo (0 to 180 µM) for a duration of 24 h. Thereafter, CCK8 was introduced, and the cells were incubated at 37 °C and 5% CO_2_ for 2 h. The absorbance at 450 nm was then measured using Synergy LX Multi-Mode Reader (BioTek Instruments, USA).

### Colony formation

8505C and KHM-5M were placed with an initial density of 2 × 10^4^ cells containing varying concentrations of Ruxo (0, 40, and 80 µM) for one week. The plates were gently washed using PBS, fixed with paraformaldehyde for 15 min, and colored with 0.1% crystal violet for 20 min.

### Transwell migration and invasion assay

The transwell cell culture chamber (Corning Costar Corp., Cambridge, MA, USA) served as the platform to assess ATC cells’ migratory and invasive capabilities. In the migration assay, cells from the 8505 C and KHM-5M lines were seeded in the upper chamber using a serum-free medium at a density of 3 × 10^4^ cells. Meanwhile, the lower chamber was loaded with RPMI-1640 medium supplemented with 10% FBS, along with varying concentrations of Ruxo (0, 40, and 80 µM). For the invasion assay, the chambers were coated with Matrigel (BD Biosciences, diluted at a 1:4 ratio) for 1 hour, followed by the subsequent steps identical to those in the migration assay. The plates were incubated at a temperature of 37 °C with a 5% CO_2_ concentration. This was maintained for 24 h for migration assessment and extended to 48 h for evaluating the invasion. Subsequently, the cells were fixed using a 4% paraformaldehyde solution for 30 min, followed by staining with 0.01% crystal violet for the same duration. The migrated and infiltrated cells were scrutinized under a microscope, and their quantity was quantified using the ImageJ software.

### Determination of cell survival rate

To determine the cell viability, an Annexin V-FITC and PI Kit (Liankebio, China) containing Annexin V-FITC and PI were employed according to the provided instructions. Plates were seeded with cells at a density of 1.5 × 10^5^ and exposed to various drug concentrations for 24 h. Afterward, the cells were harvested, PBS-washed, and stained with PI and Annexin V-FITC. The NovoCyte^®^ Quanteon^®^ Benchtop Flow Cytometer was used to obtain fluorescence data, which were then analyzed with FlowJo V10.

### Morphological examination

Morphological analyses were conducted following the methods described earlier [30]. Briefly, following the application of various substances to the cells for the specified durations, they were captured using a phase-contrast optical microscope. The observation of mitochondria involved the following steps: ATC cells were initially subjected to a specific duration of treatment with Ruxo, subsequently stained with Mito tracker for a duration of 30 min in a light-free environment, and finally examined using Confocal laser (Leica STED, Germany).

For immunofluorescence staining, the cells after treatment were subjected to a 30-minute incubation with a Mito tracker in a dark environment. Subsequently, the cells were rinsed with PBS and fixed in 4% paraformaldehyde, followed by blocking in a solution containing 3% BSA, 0.4% gelatin, and TBST. The cells were then exposed to Cyto C antibody overnight at 4 °C and washed with PBS. Following this, the cells were incubated with Alexa Fluor 488-labeled Goat Anti-Rabbit IgG(H + L) (A0423, Beyotime Biotechnology, China) for an additional hour at room temperature. Subsequently, the nuclei were subjected to staining with DAPI for a duration of 10 min, followed by examination through employment of a Confocal laser (Leica TCS SP8, Germany). The double antibody staining procedure for DRP1 and HSP60 was conducted following the above steps without Mito tracker stain. Except, Alexa Fluor 488-labeled Goat Anti-Rabbit IgG(H + L) and Alexa Fluor 555-labeled Donkey Anti-Mouse IgG(H + L) (A0460, Beyotime Biotechnology, China) were employed as secondary antibodies, and then samples were subsequently observed using a Confocal laser (Leica TCS SP8, Germany).

### Transmission electron microscopy (TEM)

The cells, specifically 8505 C, and KHM-5M, underwent an 8-hour treatment with Ruxo. After this treatment period, the cells were harvested and fixed utilizing an electron microscope fixative (HaoKe Biotechnology Co. Ltd, China). Subsequently, a rinse was performed using 0.1 M phosphate buffer (PB) with a pH of 7.4. Following this, the cells were exposed to 1% OsO_4_ in 0.1 M PB for a duration of 2 h, followed by a process of dehydration using a gradient of alcohol and 100% acetone. The prepared samples were subjected to embedding, polymerization, ultrathin slicing, and staining procedures. The images were captured using a transmission electron microscope (HT7650, HITACHI, Tokyo, Japan).

### Lactate dehydrogenase release assay (LDH)

An LDH assay (Cat: A020-1, Nanjing Jiancheng Bioengineering Institute, China) was used to determine extracellular LDH activity after various treatments according to the protocol. Briefly, after treatment, the cell culture supernatant was collected, and LDH activity was determined by following the kit procedure.

### JC-1 functional assay

The mitochondrial membrane potential was determined using JC-1 (Cat 40705ES03, Yeasen Biotechnology, China) according to the instructions provided by the manufacturer. Following a 12-hour treatment, the 8505 C and KHM-5M cells were exposed to JC-1 at a concentration of 5 µg/mL for a duration of 30 min. Subsequently, they were rinsed twice and reconstituted in PBS. Stained cells were analyzed using a NovoCyte^®^ Quanteon^®^ Benchtop Flow Cytometer.

### Intracellular ROS determination

Following an 8-hour treatment, the cells were subjected to staining using 10 µM DCFH-DA (Cat T15458, TargetMol, USA) in a dark environment for 30 min. Subsequently, the cells were rinsed twice with PBS. The cells were examined with a NovoCyte^®^ Quanteon^®^ Benchtop Flow Cytometer to ascertain ROS levels.

### siRNA transfection and CRISPR/Cas9 knock-out cell lines

Beijing Tsingke Biotech Co., Ltd., China, synthesized the siRNA that targets STAT3 and the negative control siRNA. The siRNA sequence of STAT3 was (siRNA): 5′- GGCTGGACAATATCATTGA-3′. According to the instructions provided by the manufacturer, Lipofectamine 3000 (Cat.NO. L3000015, Invitrogen) was used to transfect the siRNAs into ATC cells. After 48 h of transfection, the cells were examined using real-time quantitative PCR (RT-qPCR) and western blotting to confirm the effectiveness of siRNA knockdown.

The CRISPR/Cas9 lentivirus plasmid was constructed by Applied Biological Materials Inc., Canada. The sgRNA sequences were as follows: caspase 3: 5′-GGATGCCGGCACTACACAAC-3′, caspase 9: 5′- GAACAGCTCGCGGCTCAGCAGGG-3′, and GSDME: 5′-GGATGCCGGCACTACACAAC-3′. A lentivirus system at an MOI of 20–50 was used to infect ATC cells, followed by selecting Lenti Guide-Puro positive cells using 2–5 μg/mL puromycin. Forty-eight hours after the infection, western blot analysis was used to validate the CRISPR/Cas9 knockdown efficiency. The remaining cells were used for subsequent experiments.

### DRP1 and STAT3 overexpression

ATC cells were transfected with a DRP1 overexpression plasmid (Biocompete Inc., China) and STAT3 overexpression plasmids (Genomeditech Inc., China) using Lipofectamine 3000. After 48 h of transfection, the overexpression efficiency was validated through RT-PCR and western blotting.

### Transcription factor binding site analysis and dual-luciferase reporter assay

The Jaspar database (http://jaspar.genereg.net/about/) was used to analyze the binding sites of transcription factors within the promoter region of the human DRP1 gene Applied Biological Materials Inc., Canada, synthesized both full-length and mutated human DRP1 plasmids to construct luciferase reporters. The procedure involved cloning the wild-type (WT) full-length human/mouse DRP1 promoter sequence, Mutant 1 (deletion of ttgccaggaat), DRP1 promoter, and Mutant 2 (deletion of catcctggaaa) into pLenti-Promoterless-Dual-Luc vectors. Upon transfection, ATC cells received equitably measured DNA along with a matched empty vector. Following a 48-hour incubation, the ATC cells were isolated and subjected to assessment using the Dual-Lumi™ Luciferase Reporter Gene Assay Kit (Cat: RG088S, Beyotime Biotechnology, China), following the provided guidelines by the manufacturer.

### RT-qPCR

The RT-qPCR was conducted according to the previously mentioned protocol [31]. Briefly, total cellular RNA was extracted using the Trizol method, and PCR amplification and fluorescence real-time detection was performed after reverse transcription using PrimeScript™ RT Reagent Kit (Cat: RR037 Takara Biomedical Technology Co., Ltd., Japan). The PCR primer sequences for genes are shown in supplement table 1.

### Western blotting and antibodies

Cells were harvested employing a western blotting and IP lysis buffer containing PMSF (Cat: P0013 and ST505, Beyotime Biotechnology, China). The protein concentration was quantified using The BCA protein assay (Thermo Fisher Scientific, USA). The protein samples were subsequently separated using sodium dodecyl sulfate-polyacrylamide gel electrophoresis and transferred onto PVDF membranes. For the following steps, the membranes were blocked for an hour using a TBST solution containing 5% skim milk. Following this, the membranes were exposed to primary antibodies overnight at a temperature of 4 °C. Afterward, they were incubated with suitable HRP-conjugated secondary antibodies at a temperature of 25 °C for 60 min. Chemiluminescence was observed using a ChemiDoc-MP imager (Bio-Rad, Hercules, CA, USA) coupled with an FD Bio-Dura ECL Kit (Cat FD8020, Fdbio Science, China). The band density was analyzed using the ImageJ software. The primary antibodies used are listed in Supplement Table 2. The suitable HRP-conjugated IgG secondary antibodies, namely Goat anti-rabbit (Cat: A0208) and anti-mouse (Cat: A0216), were procured from Beyotime Institute of Biotechnology, China.

### Xenograft tumor model

The animal trials were carried out by the authorized procedure of the Animal Ethics Committee of Zhejiang Provincial People’s Hospital (Approval NO. 20221112233826207365).

Shanghai SLAC Animal Co. (Shanghai, China) provided female BALB/c nude mice, which were 3–4 weeks old. Subcutaneous implantation of 8505 C cells (5 × 10^6^ cells/100 µl) was performed on the right axilla of the mice. Upon forming palpable tumors, the mice were randomly assigned (n = 6/group). Ruxo and Mdivi-1 were diluted in 5% DMSO + 30% PEG300 + 10% Tween 80 + 55% PBS, and 100 µL of the dilute drug was administered intraperitoneally daily for 12 days. The mice were weighed regularly, and the volumes of tumors were measured every alternate day. Tumor volume was determined by applying the subsequent equation: Tumor vol (mm^3^) = 0.5 × (short diameter)^2^ × (long diameter).

The mice were euthanized after a 12-day treatment period, and their tumors, blood, and organs (liver, kidneys, and heart) were gathered. After collection, the specimens were preserved with a 4% formalin solution, followed by embedding in paraffin. Subsequently, they were sliced into sections and stained with hematoxylin and eosin (HE).

### Immunohistochemistry

Immunohistochemistry was performed as previously described [30]. The dilution ratios of the primary antibodies were as follows: JAK1, 1:100; p-JAK1, 1:100; p-STAT3, 1:250; DRP1, 1:100; c-PARP, 1:50, and c-caspase 3, 1:200. The scoring system comprises four levels, which are determined by the intensity of cellular staining. A score of 0 is assigned to samples with no positive staining (negative), while a score of 1 is given to samples exhibiting pale yellow staining (weakly positive). Furthermore, samples displaying tan staining are assigned a score of 2 (positive), while those exhibiting strongly positive tan staining receive a score of 3.

### Statistical analysis

A minimum of three repetitions were conducted for all experiments. Results are presented as average ± standard deviation or averages ± standard error of the mean. To examine the distinctions between two groups, a t-test was employed, whereas distinctions among multiple groups were assessed using either one-way ANOVA or the two-tailed unpaired Student’s t-test, followed by Bonferroni’s test. *P* value < 0.05 was considered statistically significant, with all P-values being two-sided. The analysis was conducted utilizing GraphPad Prism 9 software (USA).

## Results

### Overactivation of the JAK1/2-STAT3 pathway in ATC

**Fig. 1:**
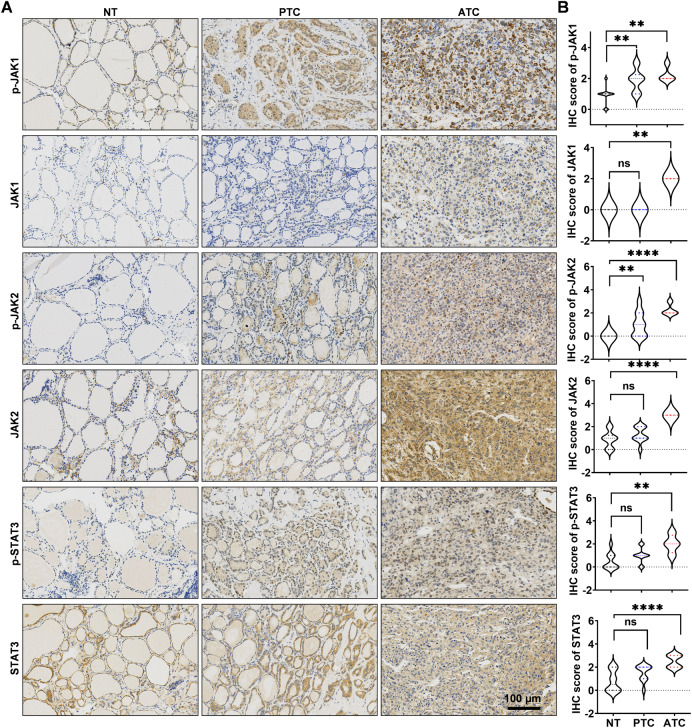
Expression of JAK1/2-STAT3 pathway from our center. **A** IHC was used to determin the expression of JAK1, p-JAK1, JAK2, p-JAK2, STAT3, p-STAT3 from our center (four ATCs, ten PTCs, and ten NT samples). **B** Violin plot of expression of p-JAK1, p-JAK2, p-STAT3, JAK1, JAK2 and STAT3, from our center. Values are presented as mean ± SD for *n* = 3, analyzed by one-way ANOVA using the Holm-Sidak method (**B**). *p < 0. 05, **p < 0. 01, ***p < 0. 001, ****p < 0. 0001.

The status whether JAK1/2-STAT3 pathway was activated in patient’s tumor tissue is considered to be an important clinical significance for Ruxo treatment [21]. In our study, we conducted IHC staining on a randomly selected set of 4 ATCs, 10 PTCs, and 10 NT tissues obtained from our center. This approach aimed to elucidate the expression of JAK1/2-STAT3. The results revealed higher levels of JAK1, JAK2, STAT3, and their phosphorylated forms within the ATC samples, compared to the levels observed in PTC and NT samples (Fig. 1). This observation suggests an excessive activation of the JAK1/2-STAT3 pathway within ATC tissues. Notably, we observed JAK1, JAK2, STAT3 were all located in ATC tumor cells, suggesting a crucial role of JAK1/2-STAT3 activation in maintaining malignant features of ATC tumor cells. We further investigated levels of JAK1, JAK2, STAT3 and their phosphorylated forms in various thyroid cell lines, including normal thyroid epithelial cell line (NTHY), three PTC cell lines (TPC-1, BCPAP, and IHH4), and six ATC cell lines (8505C, KHM-5M, C643, CAL-62 and BHT101) (Supplementary Fig. S1). Our findings indicate that the JAK1/2-STAT3 pathway was not significantly activated in normal thyroid cell line (NTHY) and BRAF^v600E^ wild type PTC cell line (TPC-1). However, it exhibited mild activation in BRAF^v600E^ mutant PTC cell lines (BCPAP, IHH4) and significant activation in ATC cell line (8505C, KHM-5M, C643, CAL-62 and BHT101). Thus, the more malignant the thyroid cancer, the higher JAK1/2-STAT3 pathway activation.

### Ruxo significantly suppresses cell viability, colony formation, cell migration, and invasion in ATC cells

**Fig. 2:**
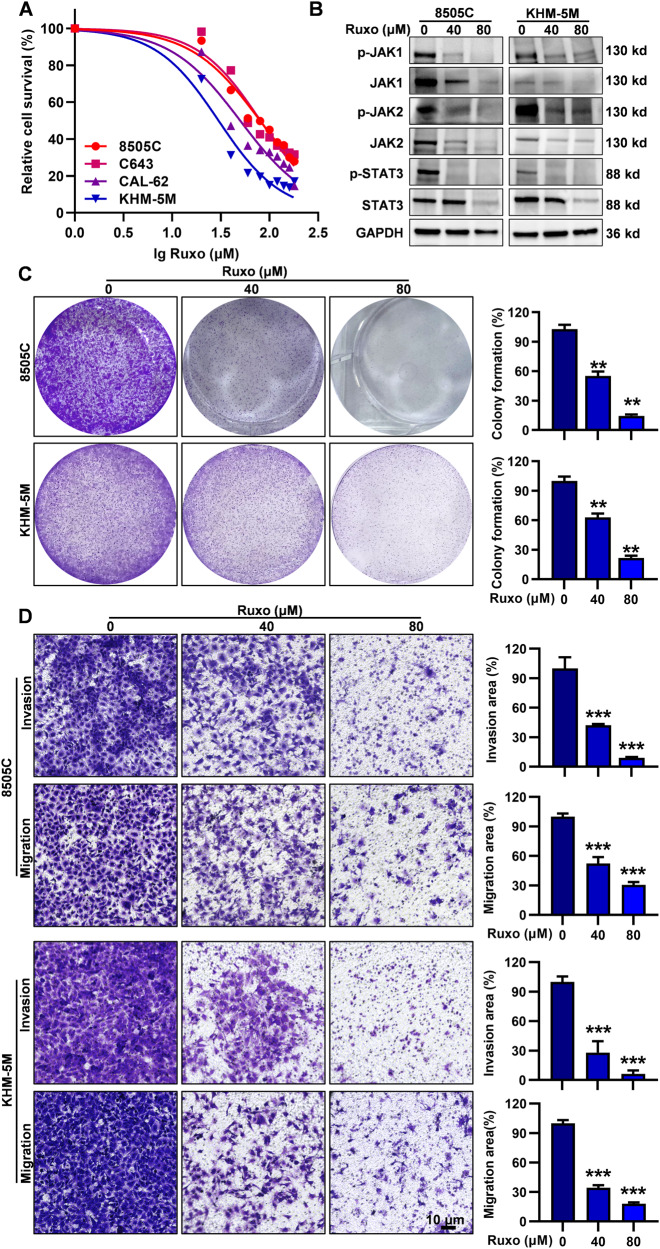
Ruxo inhibited JAK1/2-STAT3 pathway, cell viability, colony formation and cell migration and invasion in human ATC cells. **A** CCK-8 assay was used to determined cell viability of different concentrations of Ruxo (0–180 μM) to 8505C, C643, CAL-62, and KHM-5M cells after 24 h. IC_50_ values were calculated from non-linear regression plots using the GraphPad. **B** Western blot was used to determined levels of key proteins from JAK1/2-STAT3 pathway. **C** Quantitative and qualitative analyze of 8505C and KHM-5M cell clone formation at the indicated concentrations of Ruxo (*n* = 3). **D** Transwell experiments were used to investigate the migration and invasion ability changes after treatment of Ruxo. Values are presented as mean ± SD for n = 3, analyzed by one-way ANOVA using the Holm-Sidak method (**C** and **D**). *p < 0. 05, **p < 0. 01, ***p < 0. 001.

Upon confirming the overactivation of the JAK1/2-STAT3 signaling pathway within the ATC cell lines and clinical samples, we proceeded to evaluate the effects of Ruxo using various thyroid cell lines, NTHY, TPC-1, BCPAP, IHH4, 8505 C, KHM-5M, C643, CAL-62 and BHT101 in vitro. Our findings indicated that Ruxo inhibited the viability of all tested ATC cells in a dose-dependent manner. However, it has less effect on normal thyroid cells and PTC cells. The IC_50_ values of Ruxo were determined for normal thyroid epithelial cells (1008 μM), PTC (265.4-344.6 μM), and ATC (29.94-80.59 μM) as shown in Fig. 2A and Supplementary Fig. S2. To gain insights into the baseline expression of the STAT family, which includes STAT1-6, we assessed their levels. Notably, the raw Cq values for genes like STAT1, STAT2, STAT4, STAT5A, STAT5B, and STAT6 were all above 30, indicating their relatively low abundance and expression levels. In contrast, the expression of STAT3 was much higher, and at least more than 7-fold higher when compared to other members of the STAT family (Supplementary Fig. S3A). This additional analysis reinforces the notion that STAT3 is pivotal in ATC cells. In summation, our investigations underscore the significant involvement of STAT3 in the behavior of ATC cells.

We selected two doses of Ruxo based on the IC_50_, which effectively inhibited the JAK1/2-STAT3 pathway (Fig. 2B, Supplementary Fig. S3C). To further elucidate the phenomenon of reducing JAK 1/2 expression levels by Ruxo in ATC cells, we administered Ruxo (80 μM) to 8505 C and KHM-5M cells at a time-gradient dependent manner (0 h, 1 h, 2 h, 4 h, 6 h, 8 h, 12 h, 24 h) (Supplementary Fig. S3B). Notably, a significant decline in both JAK1/2 and STAT3 phosphorylated proteins was observed during the initial stages of Ruxo’s administration (1–2 h), with the p-STAT3 level being essentially undetectable at 1 h, whereas the changes in the protein levels of JAK1/2 and STAT3 occurred at 6–8 h. Furthermore, Ruxo significantly inhibited the colony formation, migration, and invasion abilities of 8505C and KHM-5M cells in a dose-dependent manner (Fig. 2C, D). We also preliminarily explored the effect of Ruxo on Epithelial-mesenchymal transition (EMT) pathway markers and found that ZEB-1, β-Catenin, N-Cadherin and Vimentin showed a significant dose-dependent decrease (Supplementary Fig. S4). The results showed that Ruxo significantly inhibited the JAK1/2-STAT3 pathway and exhibited remarkable anti-tumor activity in ATC cells.

### Ruxo-induced apoptosis and pyroptosis in ATC cells

**Fig. 3:**
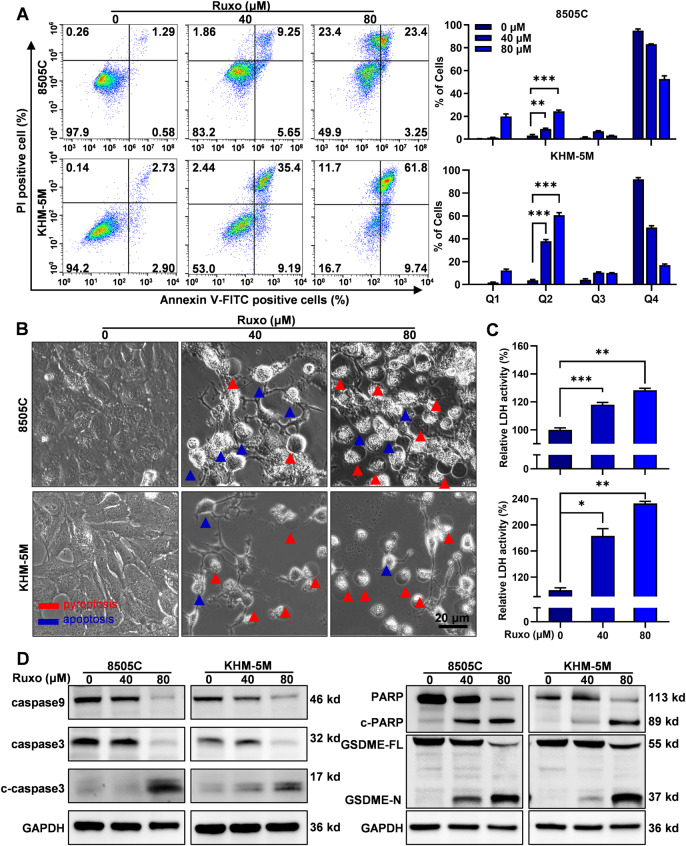
Ruxo-induced ATC cells apoptosis and pyroptosis. **A** Flow cytometry was used to measure cell deaths of 8505 C and KHM-5M after Ruxo (0, 40 and 80 μM) treatment for 24 h. **B** Characteristic morphology of 8505 C and KHM-5M cells induced by Ruxo (0, 40 and 80 μM) (apoptotic cells are shown by bule arrows while pyroptotic cells are shown by red arrows). **C** Relative LDH activities in the lysate of cells were measured by the LDH assay Kit. **D** Western blot was used to measure caspase 9, caspase 3, c-caspase 3, PARP, c-PARP, full-length GSDME and GSDME-N terminus protein levels in 8505 C and KHM-5M cells, after Ruxo (0, 40 and 80 μM) treatment for 24 h. Data are shown as mean ± SD for *n* = 3, analyzed by one-way ANOVA using the Holm-Sidak method (**A** and **C**) *p < 0. 05, **p < 0. 01, ***p < 0. 001, ****p < 0. 0001.

Ruxo can induce the death of various tumor cells, mostly by triggering apoptosis [32–35]. To explore whether Ruxo induces ATC cell death and to determine the mode of death, we stained the Ruxo-treated cells with Annexin V/PI and subjected them to flow cytometry. Ruxo significantly induced the death of ATC cells in a dose-dependent manner, and the dead cells were mainly gathered in the Q2 quadrant (Annv^+^ PI^+^), suggesting that cell death may involve simultaneous pyroptosis and apoptosis [30] (Fig. 3A). To further verify the occurrence of pyroptosis, we examined the morphological characteristics of cell death using phase-contrast optical microscopy. Cells treated with higher concentrations of Ruxo were mostly dead and swollen; the number of “balloon-like” cells increased significantly (a characteristic of pyroptosis) with increasing Ruxo concentration. Meanwhile, the number of fragmented cells and cells with “apoptotic bodies” also increased (Fig. 3B). Furthermore, our investigation revealed that Ruxo triggered the cleavage of the pyroptosis-executive molecule GSDME and the apoptosis-associated protein PARP within ATC cells (Fig. 3D, Supplementary Fig. S5A). Notably, an increase in Ruxo concentration was accompanied by a significant increase in the extracellular LDH activity, demonstrating a dose-dependent trend (Fig. 3C). Furthermore, we preliminarily explored the effect of Ruxo on necroptosis markers finding a significant dose-dependent decrease in p-RIPK1, RIPK1, p-RIP3, RIP3 and MLKL. A marginal increase in p-MLKL was observed exclusively in the 8505C cell line (Supplementary Fig. S5B). These findings indicate that the impact of Ruxo on ATC cell death is unlikely to be mediated via the necroptosis pathway. From these findings, it is apparent that Ruxo invokes both apoptosis and pyroptosis within ATC cells. Importantly, the data suggests a plausible link between Ruxo-induced pyroptosis and the cleavage of GSDME.

### Ruxo induces caspase9/3-GSDME-pyroptosis in ATC cells

Having observed obvious GSDME cleavage, we employed the CRISPR/Cas9 system to target GSDME in ATC cells. The findings suggested that GSDME knock-out significantly reduced Ruxo-induced pyroptosis and extracellular LDH activities (Supplementary Fig. S7), this collectively point to the conclusion that Ruxo ‘s induction of pyroptosis within ATC cells mainly through the GSDME-dependent pathway.

**Fig. 4:**
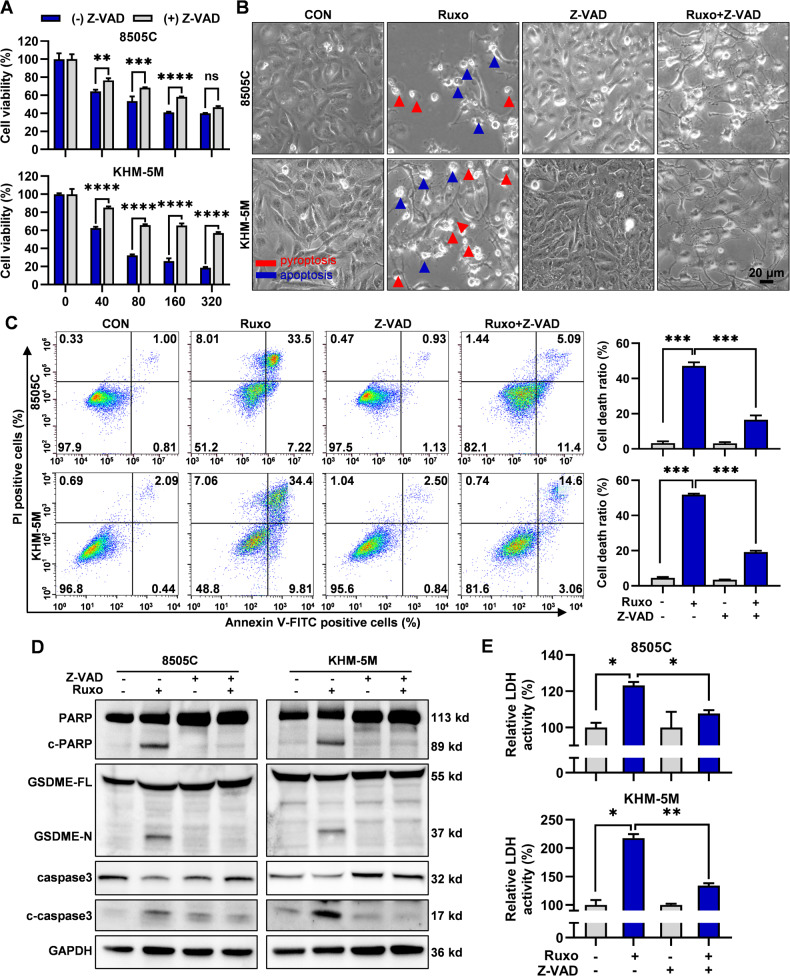
Ruxo induced caspase-dependent pathways apoptosis and pyroptosis in human ATC cells. **A** Z-VAD (40 μM) was added into the plates 2 h before treatment of Ruxo. CCK-8 assay were used to determine the cell viabilities of 8505C and KHM-5M cells exposed to Ruxo (0, 40, 80, 160 and 320 μM) for 24 h, with or without Z-VAD. **B** Characteristic morphology of Ruxo treated ATC cells (8505C and KHM-5M, 40 μM, for 24 h) are shown, with or without Z-VAD, **C** Cell deaths of Ruxo treated ATC cells (8505C, 80 μM and KHM-5M, 40 μM, for 24 h) was measured by flow cytometry and the ratio presented. **D** Western blot was used to measure caspase 3, c-caspase 3, PARP, c-PARP, full-length GSDME and GSDME-N terminus protein levels in 8505C and KHM-5M cells after Ruxo (0, 40 μM) treatment for 24 h, with or without Z-VAD. **E** Relative LDH activities in the lysate of cells were measured by the LDH assay Kit. Data are shown as mean ± SD for *n* = 3, analyzed by one-way ANOVA using the Holm-Sidak method (**A**), or two-way ANOVA using the Tukey method (**C** and **E**) for multiple comparisons. *p < 0. 05, **p < 0. 01, ***p < 0. 001, ****p < 0. 0001.

Caspase 3 is the main executor involved in the cleavage of GSDME of the caspase family, and caspase 8 and 9, upstream of caspase 3, represent the mitochondria-independent and dependent cell death pathways, respectively [28]. We observed that Ruxo induced cleavage of caspase 9 and 3 in a dose-dependent manner (Fig. 3D). When combined with a broad-spectrum caspase inhibitor (Z-VAD-FMK), we found Ruxo-induced pyroptosis and apoptosis were significantly attenuated with decreased cell death ratio, the cleavage of GSDME and PARP, and the release of LDH. This was in line with the cell death ratio analysis results by flow cytometry, the cleavage of GSDME and PARP, and the release of LDH (Fig. 4, Supplementary Fig. S6). Furthermore, by the introduction of the CRISPR/Cas9 system, we generated caspase 9 knock out, and caspase 3 knock out ATC cells, respectively. Both caspase 9 and 3 knock-out in ATC cells significantly reduced Ruxo-induced pyroptosis and extracellular LDH activities (Supplementary Fig. S7). These results show that Ruxo induces pyroptosis in ATC cells via a caspase 9/3-GSDME-dependent pathway, which may be related to the mitochondrial pathway.

### ATC cell death induced by Ruxo is associated with mitochondrial dysfunction

**Fig. 5:**
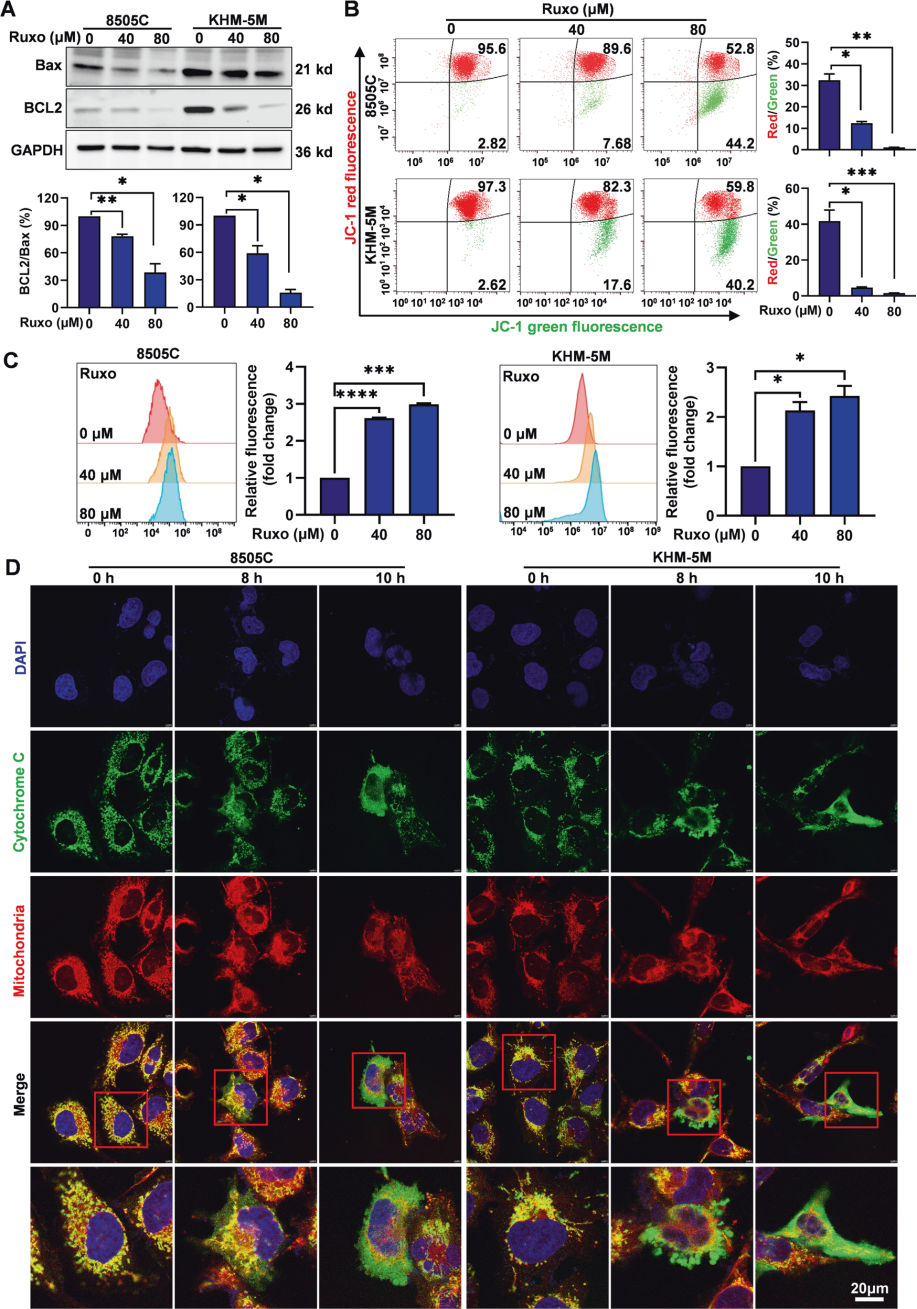
ATC cell death induced by Ruxo is associated with dysfunction of mitochondrial. **A** Western blot was used to evaluate levels of Bcl-2 and Bax protein in Ruxo-treated ATC cells (8505 C and KHM-5M, 0, 40, and 80 μM, for 24 h). Quantitative analysis of Bcl-2 and Bax by densitometry are shown as Bcl-2/Bax ratio. **B** Flow cytometry was used to determine the distribution ratios of red and green cell populations of Ruxo-treated ATC cells (8505 C and KHM-5M, 0, 40, and 80 μM, for 12 h). **C** Flow cytometry was used to determine the ROS level intracellular 8505 C and KHM-5M treated with Ruxo (0, 40 and 80 μM) for 8 h. Histograms of corresponding mean fluorescence intensity are shown. **D** Confocal laser was used to observe the co-localizations of Cyto C with the mitochondria in 8505 C and KHM-5M cells treated with Ruxo (0, 40 μM for 8 h and 10 h). Data are shown as mean ± SD for *n* = 3, analyzed by one-way ANOVA using the Holm-Sidak method (A-C). *p < 0. 05, **p < 0. 01, ***p < 0. 001, ****p < 0. 0001.

The clue that Ruxo mediated caspase 9/3-dependent apoptosis and GSDME-pyroptosis in ATC cells suggested the intrinsic cell death pathway (also known as the mitochondrial pathway) was involved in the ATC cell death. We first observed Ruxo significantly downregulated anti-apoptotic protein Bcl-2, with a significantly decreased Bcl-2/Bax ratio (Fig. 5A). By using a mitochondrial membrane potential (ΔΨm) probe (JC-1) combined and a ROS probe (DCFH-DA) with flow cytometry, we found that with increased Ruxo doses, the ΔΨm of ATC cells gradually decreased (Fig. 5B) along with apparently increased ROS generation (Fig. 5C), which further support indicated mitochondrial cell death pathway was involved. Cyto C release from mitochondria is a critical event in the mitochondrial cell death pathway. Through the confocal laser analysis, we observed obvious translocation of Cyto C from the mitochondria to the cytoplasm in ATC cells after Ruxo treatment (Fig. 5D). These results suggested that mitochondrial dysfunction triggered the mitochondrial Cyto C release pathway to mediate Ruxo-induced pyroptosis and apoptosis in ATC cells.

### Ruxo disrupts mitochondrial fission by transcription inhibition of DRP1

Mitochondria are important “energy factories” in cells and participate in the regulation of calcium homeostasis, cell cycle, apoptosis, and immune signaling pathways [36].

**Fig. 6:**
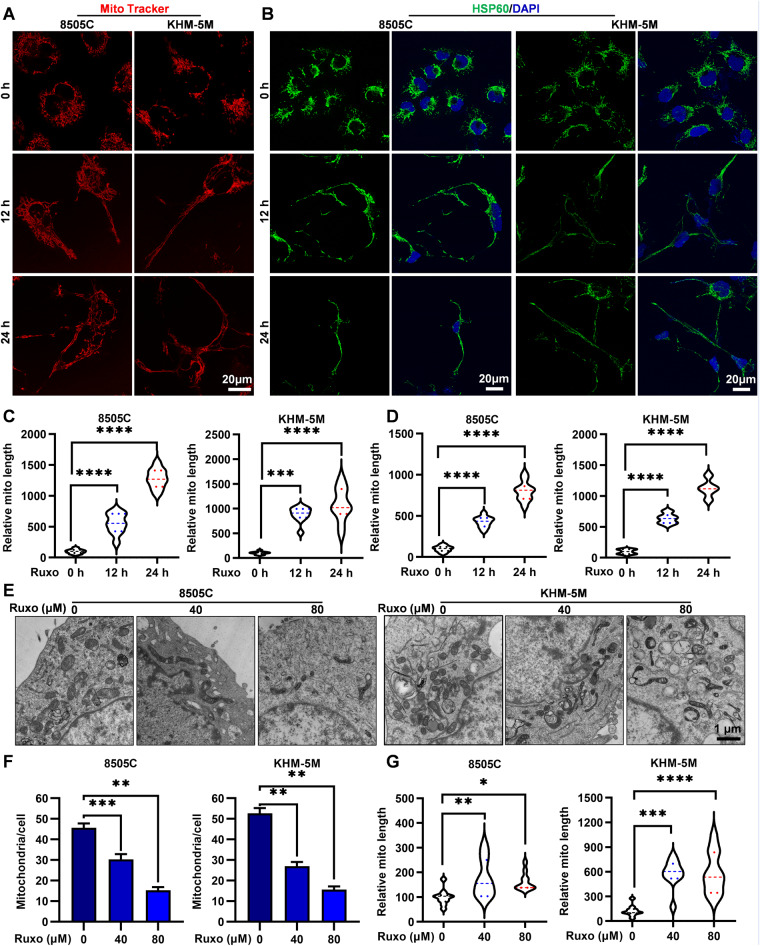
Ruxo induces mitochondrial fission disorder in ATC cell. **A** Confocal laser (Leica STED, Germany) was used to observe mitochondrial morphological changes in 8505C and KHM-5M cells treated with Ruxo (0, 40 μM for 0 h, 12 h, 24 h), used Mito tracker Deep Red FM. **B** Confocal laser was used to observe mitochondrial morphological changes in 8505C and KHM-5M cells treated with Ruxo (0, 40 μM for 0 h, 12 h, 24 h), after incubated with HSP60 antibody by immunofluorescence. **C**, **D** Quantification of mitochondrial length of **A** and **B**. **E** Transmission electron microscopy was used to observe mitochondrial morphological changes in 8505C and KHM-5M cells treated with Ruxo (0, 40, 80 μM for 8 h). **F**, **G** Quantification of mitochondrial number and length of C. Data are shown as mean ± SD for *n* = 3, analyzed by one-way ANOVA using the Holm-Sidak method (**C**, **D**, **F**, **G**). *p < 0. 05, **p < 0. 01, ***p < 0. 001, ****p < 0. 0001.

We employed a mitochondrial specific probe (Mito tracker) dependent confocal laser analysis. Obviously, the elongated mitochondria in 8505 C and KHM-5M were clearly visible, under the increased time of exposure to Ruxo (Fig. 6A, C). In order to mitigate the potential impact of probe toxicity, we employed immunofluorescence analysis to label mitochondria with HSP60 antibody. Subsequently, we also observed a significant elongation of mitochondria in ATC cells following prolonged exposure to Ruxo (Fig. 6B, D). This trend was consistently supported by electron microscopy analysis, which not only reaffirmed the decline in the count of mitochondria induced by Ruxo but also highlighted the prevalent occurrence of elongated mitochondrial shapes (Fig. 6E, F and G, Supplementary Fig. S8A).

**Fig. 7:**
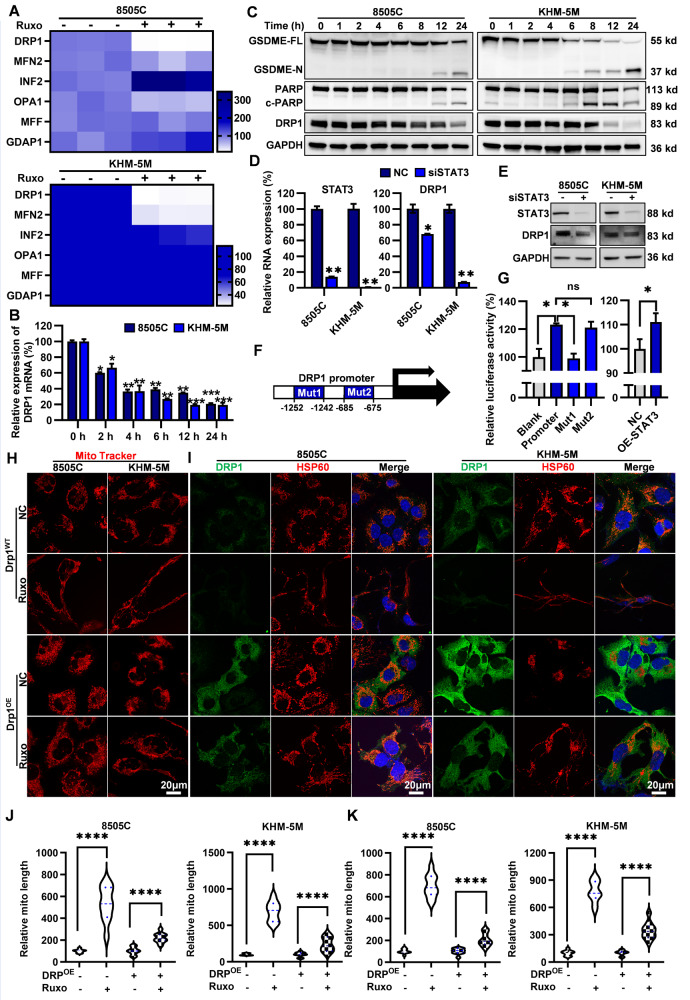
Ruxo downregulates DRP1 through inhibition of JAK1/2-STAT3 signaling pathway activation and causes mitochondrial division dysfunction. **A** RT-PCR were used to determinate the levels of mitochondrial fission-related genes, after treating with Ruxo (0, and 80 μM) for 24 h. **B**, **C** RT-PCR were used to measure the DRP1 RNA at various time intervals (0, 2 h, 4 h, 6 h, 8 h, 12 h, 24 h) with treatment of Ruxo (80 μM). And western blot was used to determinate the protein levels of DRP1, PARP, c-PARP, full-length GSDME and GSDME-N terminus. **D**, **E** RNA and protein change of STAT3 and DRP1 were measure after transfection of si-STAT3 by RT-PCR and western blot. **F**, **G** Schematic diagram of DRP1 promoter containing two putative STAT3 binding sites. STAT3 transcriptional regulation of DRP1 luciferase reporter gene assay. **H** After transfected with DRP1 overexpression plasmid for 48 h, confocal laser (Leica STED, Germany) was used to observe mitochondrial morphological changes in 8505C and KHM-5M cells, treated with Ruxo (0, 40 μM for 0 h, 12 h), where mitochondrial were stained by Mito tracker Deep Red FM. **I** After transfected with DRP1 overexpression plasmid for 48 h, confocal laser was used to observe mitochondrial morphological changes in 8505C and KHM-5M cells, treated with Ruxo (0, 40 μM for 0 h, 12 h), where mitochondrial were stained by HSP60 antibody. **J**, **K** Quantification of mitochondrial length of **H** and **I**. Data are shown as mean ± SD for *n* = 3, analyzed by one-way ANOVA using the Holm-Sidak method (**B**, **D**, **I**, **J**), or two-way ANOVA using the Tukey method (**G**) for multiple comparisons. *p < 0. 05, **p < 0. 01, ***p < 0. 001, ****p < 0. 0001.

These observations strongly suggest the possibility of a hindered mitochondrial fission process. Mitochondrial Dynamics, mitochondrial fusion and division, control the number and shape of mitochondria. Moreover, they actively contribute to the modulation of mitochondrial dysfunction and cellular demise [37]. Therefore, we investigated the key factors involved in regulating mitochondrial fusion and fission [38] and focused on changes in the expression of related genes after Ruxo treatment. RT-qPCR analysis indicates that Ruxo significantly downregulated the mRNA expression of DRP1 and MFN2 in 8505 C and KHM-5M cells in a dose-dependent manner, with the maximum reduction of DRP1. Meanwhile, Ruxo significantly increased mRNA level of INF2 and OPA1, whereas no obvious changes in MFF and GDAP1 (Fig. 7A). Furthermore, the authors verified the protein level expression of six mitochondrial fission and fusion markers (DRP1, MFN2, INF2, OPA1, MFF and GDAP1) by Western Blot (Supplementary Fig. S8B). Additionally, a heat map has been generated to illustrate the alterations in protein level expression of these six genes in ATC before and after Ruxo treatment, as depicted in Supplementary Fig. S8B. Notably, the expression levels of the six genes demonstrated a downward trend at the protein level, with exception of the OPA1 protein level in 8505C, where the down-regulation of DRP1 was particularly pronounced, aligning with the RNA level.

Among the six genes, MFN2 and OPA1 are genes associated with mitochondrial fusion [39]. The findings of our study indicate that MFN2 and OPA1, exhibits a declining trend in ATC cells upon exposure to Ruxo. Reduction in mitochondrial fusion may lead to shorter and more abundant mitochondria, if alterations in mitochondrial fusion genes significantly contribute to the pharmacological effects of Ruxo. However, this observation contradicts the observed phenotype of elongated and fewer mitochondria, suggesting that the decrease in mitochondrial fusion may not be the primary determinant. DRP1, INF2, and MFF are genes associated with mitochondrial fission. Among these genes, INF2 primarily interacts with DRP1 to facilitate actin recruitment [40], while MFF serves as the primary receptor for DRP1 [41]. Notably, DRP1 appears to exert a dominant influence on the process of mitochondrial division [42]. Our experimental findings further support this notion, as the down-regulation of DRP1 was most pronounced at both the RNA and protein levels. Consequently, we have selected DRP1 as the primary gene of our study. Then we verified the effect of Ruxo on both mRNA and protein levels of DRP1 in 8505 C and KHM-5M cells and found that Ruxo downregulated the mRNA and protein levels of DRP1 in a time-dependent manner, accompanied by the cleavage of PARP and GSDME (Fig. 7C, Supplementary Fig. S8C). These results indicated that Ruxo induced mitochondrial fission disorder mainly depended on the suppression of DRP1 transcription.

### STAT3 directly regulated the transactivation of DRP1 expression

Repression of DRP1 transcription by Ruxo in ATC cells gave us a great clue that STAT3, a major downstream transcription factor, may directly regulate the transcription of DRP1. Meanwhile, exceptionally abundant mRNA of STAT3 foreboded that STAT3 may serve as a main transcriptional regulator of DRP1 in ATC cells (Supplementary Fig. S3A). Subsequently, we knocked down STAT3 and found that the expression of DRP1 in 8505 C and KHM-5M cells decreased synchronously, as expected (Fig. 7D, E). This was also confirmed by a STAT3 inhibitor STX-0119, with significant inhibition of STAT3 phosphorylation and downregulation of both DRP1 mRNA and protein levels in a dose-dependent manner (Supplementary Fig. S9C). These results suggested that DRP1 might be a direct target gene of STAT3. Using JASPAR, we identified potential binding sites for STAT3 in the promoter region (position) of DRP1 (as indicated in Supplementary Table 3). Through transfection of a dual-luciferase reporter system with both wild-type and mutated promoter-binding regions into ATC cells, a noticeable reduction in luminescence signal was observed from the mutated promoter. Furthermore, upon overexpressing STAT3, there was a substantial increase in the luminescence intensity of the wild-type promoter. These findings strongly imply that STAT3 potentially engages in direct binding to DRP1 and exerts transcriptional control (Fig. 7F, G). Moreover, DRP1 was significantly highly expressed in ATC patients compared to PTC and NC patients (Supplementary Fig. S9A). Meanwhile, to reconfirm whether overexpression of DRP1 restores mitochondrial damage by Ruxo. DRP1 was overexpressed in ATC cells using a plasmid for a duration of 48 h. Subsequently, the cells were subjected to a 12 h treatment with 40 uM Ruxo. After staining with Mito tracker or performing immunofluorescence co-staining with HSP60 and DRP1 antibodies, we observed consistent results indicating that increased DRP1 expression restored mitochondrial morphology in ATC cells treated with Ruxo (Fig. 7H–K). Take together, these results propose a direct transcriptional role of STAT3 in upregulating DRP1 expression, thereby facilitating mitochondrial fission within ATC cells. Inhibiting the JAK1/2-STAT3 pathway affects DRP1 transcription and downregulates DRP1 protein expression, resulting in mitochondrial division disorders leading to cell death in ATC cells.

### DRP1 expression crucially contributes to Ruxo-induced apoptosis and pyroptosis in ATC cells

**Fig. 8:**
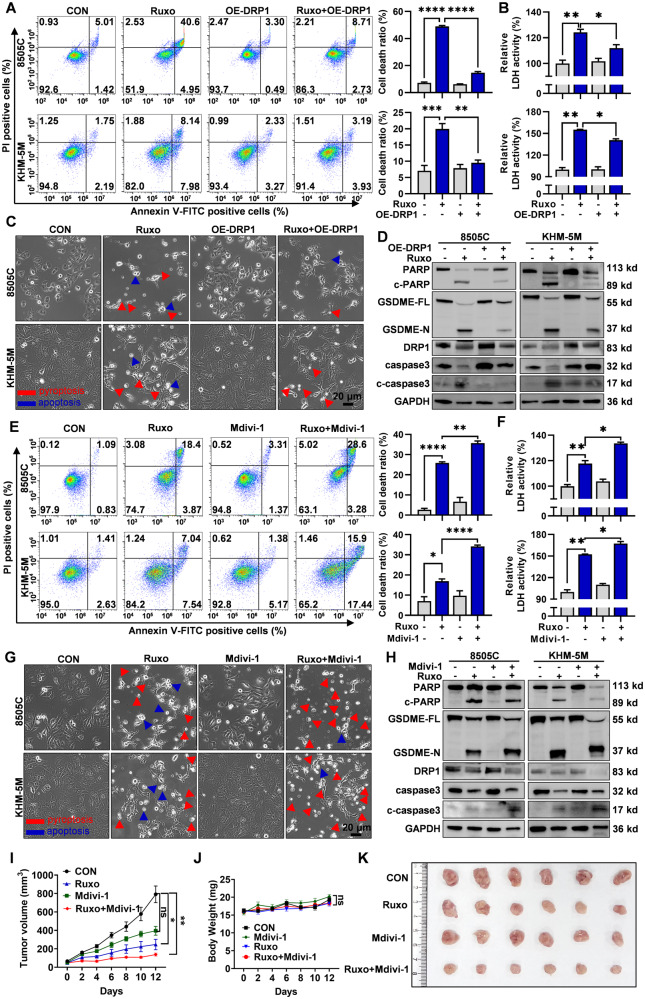
DRP1 expression is closely associated with Ruxo-induced ATC cell death. Cell deaths (**A**), relative LDH activities in culture mediums (**B**), morphological alterations (**C**) and DRP1, caspase 3, c-caspase 3, PARP, c-PARP, full-length GSDME and GSDME-N terminus protein levels (**D**) of Ruxo -treated ATC cells (8505C, 40 μM and KHM-5M, 20 μM, for 24 h) with or without transfection of DRP1 overexpression plasmid were measured by flow cytometry, LDH assay and western blot. Cell deaths (**E**), relative LDH activities in culture mediums (**F**), morphological alterations (**G**) and DRP1, caspase 3, c-caspase 3, PARP, c-PARP, full-length GSDME and GSDME-N terminus protein levels (**H**) of ATC cells treated with or without Ruxo (8505C, 40 μM and KHM-5M, 20 μM, for 24 h) and Mdivi-1 (a DRP1 inhibitor), were measured by flow cytometry, LDH assay and western blot. (**I** and **J**) Tumor volumes were measured every other day, growth curves of tumors treated with Ruxo (15 mg/kg, intraperitoneal injection), Mdivi-1 (15 mg/kg, intraperitoneal injection) and Ruxo (15 mg/kg) +Mdivi-1 (15 mg/kg) (n = 6/group) were analyzed by GraphPad Prism 9 software. Data are shown as mean ± SD. **K** Representative gross image of tumors at the endpoint of the experiment were performed of each group. Data are shown as mean ± SD for *n* = 3, analyzed by two-way ANOVA using the Tukey method (**A**, **B**, **E**, **F**) for multiple comparisons. *p < 0. 05, **p < 0. 01, ***p < 0. 001, ****p < 0. 0001.

We confirmed that DRP1 is a downstream target directly regulated by JAK1/2-STAT3. It is necessary to further clarify the role of DRP1 in the Ruxo-induced ATC mitochondrial cell death pathway. First, ATC cells overexpressing DRP1 clearly showed recovery in cell viability, decreased cell death (Fig. 8A, C, Supplementary Fig. S10B), significantly reduced release of LDH, and reduced cleavage of PARP, GSDME, and caspase 3 when treated with Ruxo (Fig. 8B, D, Supplementary Fig. S10A). In contrast, we cotreated the cells with Ruxo and a DRP1 inhibitor, Mdivi-1, and found that Mdivi-1 can act synergistically with Ruxo to inhibit the viability of ATC cells and induce cell death (Fig. 8E). Further, most cells died through pyroptosis (Fig. 8G), and an increased release of LDH and cleavage of PARP, GSDME, and caspase 3 were observed (Fig. 8F, H, Supplementary Fig. S10C). Furthermore, in vivo, a significant tumor inhibition was observed in the combined Ruxo and Mdivi-1 group. While a noticeable decrease in tumor inhibition rate was observed in the low-dose Ruxo group alone not Mdivi-1 group. These findings align with the outcomes of the in vivo experiments (Fig. 8I, J, K). Taken together, these findings suggest that DRP1 is a key target of the Ruxo-mediated inhibition of JAK1/2-STAT3-induced pyroptosis and apoptosis in ATC cells.

### Ruxo inhibits the growth of mice xenografts in vivo

**Fig. 9:**
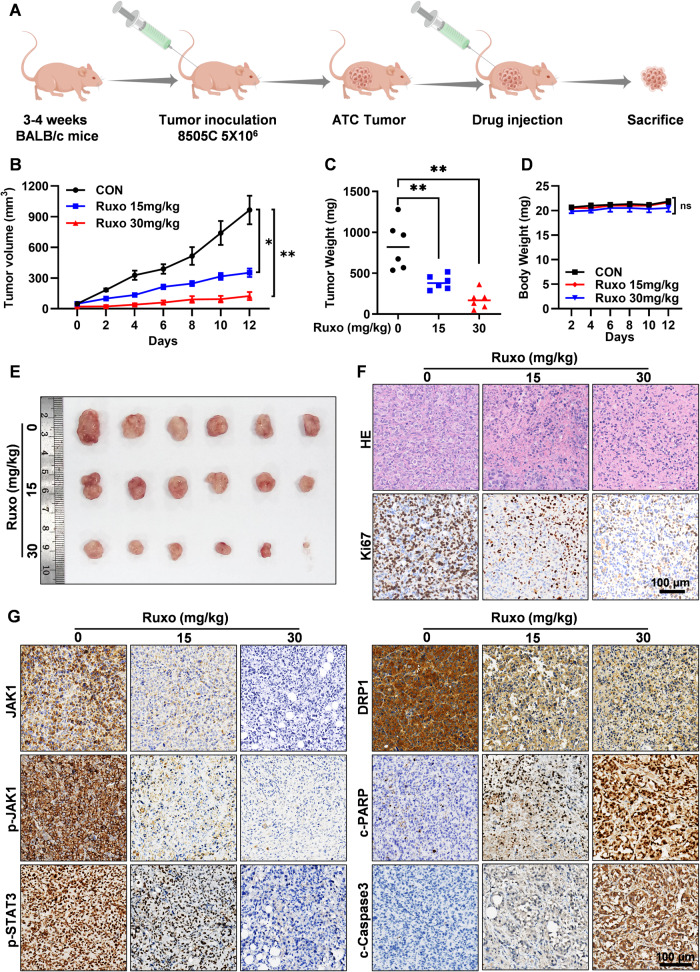
Ruxo inhibited the growth of mice xenografts in vivo. **A** Schematic diagram of animal experiments. **B** Tumor volumes were measured every other day, growth curves of tumors treated with Ruxo (0, 15 and 30 mg/kg, intraperitoneal injection) (*n* = 6/group) were analyzed by GraphPad Prism 9 software. Data are shown as mean ± SD. **C**, **D** Bar charts showed average tumor weights and body weights of each group. Data are shown as mean ± SD (*n* = 6). **E** Representative gross image of tumors at the endpoint of the experiment. **F** HE staining and Ki67 by IHC staining assay was performed of each group. **G** JAK1, p-JAK1, p-STAT3, DRP1, c-PARP, c-caspase 3, expression levels in tumors as measured by IHC staining assay. Values are presented as mean ± SD for *n* = 3, analyzed by one-way ANOVA using the Holm-Sidak method (B-D). *p < 0. 05, **p < 0. 01.

Our findings have demonstrated that Ruxo exerts an inhibitory effect on the JAK1/2-STAT3 in vitro. This inhibition, in turn, disrupts DRP1 transcription, leading to the initiation of pyroptosis and apoptosis processes within ATC cells. With these observations in mind, we proceeded to employ 8505 C cells to construct a subcutaneous xenograft tumor model in female BALB/c nude mice to evaluate the efficacy of Ruxo treatment in vivo. After tumor formation, the mice were randomly divided into groups. These groups were subsequently subjected to intraperitoneal injections with varying doses of Ruxo: 0 mg/kg (control group), 15 mg/kg, or 30 mg/kg (Fig. 9A). Compared to the control group, Ruxo (15 and 30 mg/kg) significantly inhibited the growth of tumors in mice in a dose-dependent manner (Fig. 9B, C and E) without significantly affecting body weight (Fig. 9D). HE staining of the heart, liver, and kidney tissues in each group showed no pathological changes after Ruxo administration (Supplementary Fig. S11A), and there was no significant difference in liver and renal function (Supplementary Fig. S11B). In addition, IHC staining showed that the levels of c-caspase 3 and c-PARP were increased in the Ruxo treatment group, whereas the levels of JAK1, p-JAK1, p-STAT3, DRP1, and Ki-67 were reduced (Fig. 9F, G). These results are consistent with the results of the in vitro experiments, which indicated that Ruxo could inhibit the growth of ATC tumors and induce pyroptosis and apoptosis of ATC cells without obvious drug toxicity.

## Discussion

ATC exhibits a low incidence rate among malignant tumors; however, it is characterized by rapid progression and boasts the most unfavorable prognosis among all thyroid cancers. Significantly, a notable proportion of ATC patients (15% to 50%) are diagnosed at advanced stages, rendering the treatment window exceedingly narrow [4]. Postoperative local recurrence and distant metastases occur frequently [4]. The standard treatment for ATC involves the combination of Doxorubicin and radiotherapy, a strategy that triggers tumor cell apoptosis. Despite its effectiveness, this approach is susceptible to drug resistance and has significant side effects. While the FDA has approved the use of dabrafenib and trametinib in combination therapy for ATC cases featuring the BRAF^V600E^ mutation, a substantial gap remains for the approximately 50% of ATC patients lacking such gene mutations. Moreover, it is important to acknowledge that targeted drugs, though promising, carry an inherent risk of inducing drug resistance [43]. Therefore, new targets and drugs for treating ATC are urgently required.

In various tumors, including colorectal, lung, and breast cancers [35], JAK1/2-STAT3 is overactivated. It transcriptionally activates a series of target genes, promoting tumor cell proliferation, survival, and metastasis while inhibiting immune cell infiltration [44]. In ATC, it has been observed that Glioma-associated oncogene antagonist-61 [45] and resveratrol [46] possess the potential to elicit anti-tumor effects through the inhibition of the JAK/STAT pathway. Whole-genome sequencing and whole-exome sequencing analysis of ATC have shown that JAK2/3-STAT1/2 mRNA levels are highly upregulated in ATC, suggesting an overactivated state of JAK2/3-STAT1/2 [47]. JAK1/2-STAT1/3 is compensatorily upregulated under the intervention of BRAF^V600E^ inhibitors and is involved in developing drug resistance in BRAF^V600E^ ATC [48]. Surprisingly, it appears that the analysis of JAK1/2-STAT3, along with their phosphorylated forms, within ATC patient tumor tissues has been relatively infrequent. In our current study, we gathered clinical samples of ATC, PTC, and NT tissues and established a noteworthy confirmation of the heightened expression levels of JAK1, p-JAK1, JAK2, p-JAK2, STAT3, and p-STAT3 proteins within the tumor cells of ATC patients. Furthermore, through examination of the expression of JAK1, p-JAK1, JAK2, p-JAK2, STAT3, and p-STAT3 in diverse thyroid cancer cell lines, it was discovered that ATC cells exhibit elevated levels of background expression. This robust finding strongly suggests the involvement of JAK1/2-STAT3 pathway overactivation in the progression of ATC. Importantly, to our knowledge, this study marks the first instance in which we present a molecular basis for the potential of JAK1/2-STAT3 targeted therapy in treating ATC.

Ruxo, the first kinase inhibitor for non-tumor indications, was approved in 2011 for treating myelofibrosis [12]. Today, Ruxo has been extensively introduced into both preclinical and clinical studies of a wide range of tumor types, and its promising therapeutic effects have been revealed [15]; these include cell cycle arrest [35], cell proliferation inhibition [49], cellular senescence [50], inhibition of two major DNA double-strand break repair mechanisms [51], and induction of apoptosis [52]. However, the mechanism underlying the anti-ATC effects of Ruxo and its action has yet to be fully elucidated. Our findings have demonstrated that Ruxo significantly impedes the proliferation, colony formation, invasion, and migration of ATC cells. Moreover, it induces apoptosis and pyroptosis via a mitochondria-dependent route. Remarkably, the consistent results extend to the inhibition of subcutaneous xenograft ATC tumor growth while maintaining a high level of safety. Hereby, we first found that pyroptosis, through the pathway of mitochondria-dependent caspase 3 activation mediated cleavage of GSDME, was involved in anti-ATC pharmacological process of Ruxo. Notably, pyroptosis is emerging as a prospective strategy in tumor treatment due to its classification as an immunogenic form of cell death. This attribute holds immense potential for enhancing the effectiveness of anti-cancer immunotherapy. Ruxolitinib reportedly inhibited STAT3 activity in pancreatic cancer cells, leading to reversing tumor-mediated immune suppression to enhance T cell activation; however, whether pyroptosis of the pancreatic cancer cells occurred was uncertain [44, 53]. Therefore, we postulated that pyroptosis of ATC cells induced by Ruxo would reverse the immunosuppressive microenvironment to further suppress tumor progression, providing a new avenue for combined immunotherapy. However, immune-competent mouse ATC tumor models should be established to assess the anti-ATC effect of Ruxo concerning immune response activation.

The mitochondrial regulation of apoptosis is well-known, especially with regard to the induction of apoptosis in tumor cells [54]. Recent studies have focused on the role of mitochondrial dynamics, i.e., mitochondrial fission and fusion, in apoptosis induction. In the early stages of apoptosis, the mitochondrial structure is fragmented, and mitochondrial fission and fusion-related molecules participate in apoptosis regulation. Because apoptosis and pyroptosis share the caspase pathway, it has been reported that the mitochondrial pathway participates in the regulation of apoptosis and pyroptosis. Yet, there remains a lack of consensus regarding the mechanisms by how mitochondrial fission and fusion disorder triggers apoptosis and pyroptosis. In the apoptosis-related research, majority of works suggest that upregulation of DRP1 expression could lead to aberrant mitochondrial fission resulted apoptosis [55, 56]. Conversely, a subset of literature proposes that apoptosis can be induced by inhibiting of DRP1 [57]. Additionally, it has been reported that inhibiting DRP1 results in decreased proliferation and increases apoptosis in lung cancer cells [58]. Notably, within the realm of pyroptosis investigations, paper currently reporting an increase in DRP1 could cause an accumulation of intracellular ROS which induces pyroptosis through the NLRP3/caspase1/GSDMD pathway [59]. In the present study, we observed that Ruxo significantly downregulated the expression of DRP1 in ATC cells, causing mitochondrial fission disorder, which in turn caused mitochondrial damage-mediated apoptosis and GSDME-dependent pyroptosis. For the first time, this demonstrated that DRP1 directly participates in the regulation of pyroptosis caused by mitochondrial fission disorders. To some extent, mitochondrial fission mediated by the stable expression of DRP1 may be an important basis for the rapid proliferation of tumor cells, especially ATC.

In addition, with regard to the pharmacological effects of Ruxo, we observed that JAK1/2-STAT3 plays a key role in maintaining the expression of DRP1, reflecting that STAT3 can directly regulate DRP1 expression. Intriguingly, Atractylolide III reportedly reduced DRP1 phosphorylation and mitochondrial translocation by inhibiting JAK2/STAT3, inhibiting mitochondrial fission, and improving cerebral ischemic injury and neuroinflammation [60], which differs from our results. Therefore, our study reveals a novel mechanism by which STAT3 directly regulates mitochondrial fission and offers a molecular mechanism whereby overactivation of JAK1/2-STAT3 mediates high-level expression of DRP1 to maintain a high ratio of mitochondrial fission, which may support the rapid proliferation of ATC cells. However, the functional role of DRP1 and the role of mitochondrial fission and fusion in ATC progression needs to be explored.

## Conclusion

In the current study, we not only confirmed the overactivation of the JAK1/2-STAT3 signaling pathway in ATC patients but also unveiled compelling anti-ATC effects exerted by Ruxo across both in vitro and in vivo models while ensuring a high level of safety. This study, in fact, represents the pioneer attempt to showcase Ruxo’s suppression of JAK1/2 activity, a phenomenon that results in the inhibition of STAT3-mediated transactivation of DRP1. This inhibition subsequently leads to a deficiency in mitochondrial fission, followed by the activation of caspase-dependent apoptosis and pyroptosis. The findings highlight novel mechanisms of the anti-cancer pharmacological effects of Ruxo, and favor Ruxo as a potential therapeutic drug for ATC treatment.

### Supplementary information

aj-checklist

Supplement figure

supplement table

Original Data File

## Data Availability

The corresponding author can provide the data supporting the findings of this study upon reasonable request.
