# Supplementary material for: Ruxolitinib induces apoptosis and pyroptosis of anaplastic thyroid cancer via the transcriptional inhibition of DRP1-mediated mitochondrial fission
Source: Cell Death Dis. 2024 Feb 9;15(2):125. doi: 10.1038/s41419-024-06511-1 (PMC10858168; doi:10.1038/s41419-024-06511-1)
Supplement: Supplementary file 2 — Supplement figure [file 41419_2024_6511_MOESM2_ESM.docx]

**Supplement figures**


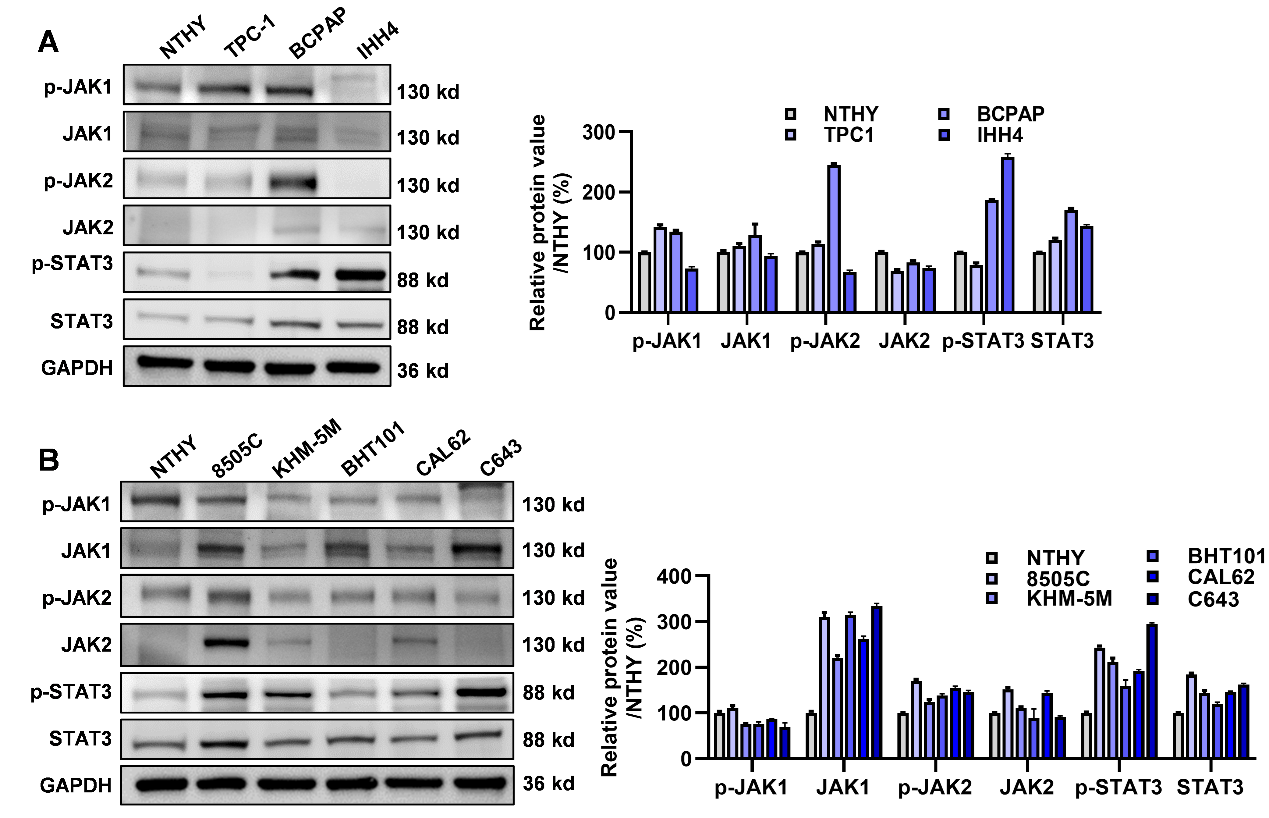


**Figure S1.** Expression of JAK1/2-STAT3 pathway genes in thyroid cell lines. (A) Quantitative and qualitative analyze of protein levels from JAK1/2-STAT3 pathway by western blot assays in normal thyroid epithelial cells (NTHY), papillary thyroid cancer cell (TPC-1, BCPAP, IHH4). (B) Quantitative and qualitative analyze of protein levels from JAK1/2-STAT3 pathway by western blot assays in NTHY and ATC cells (8505C, KHM-5M, BHT101, CAL62, C643). Values are presented as mean ± SD for n = 3.


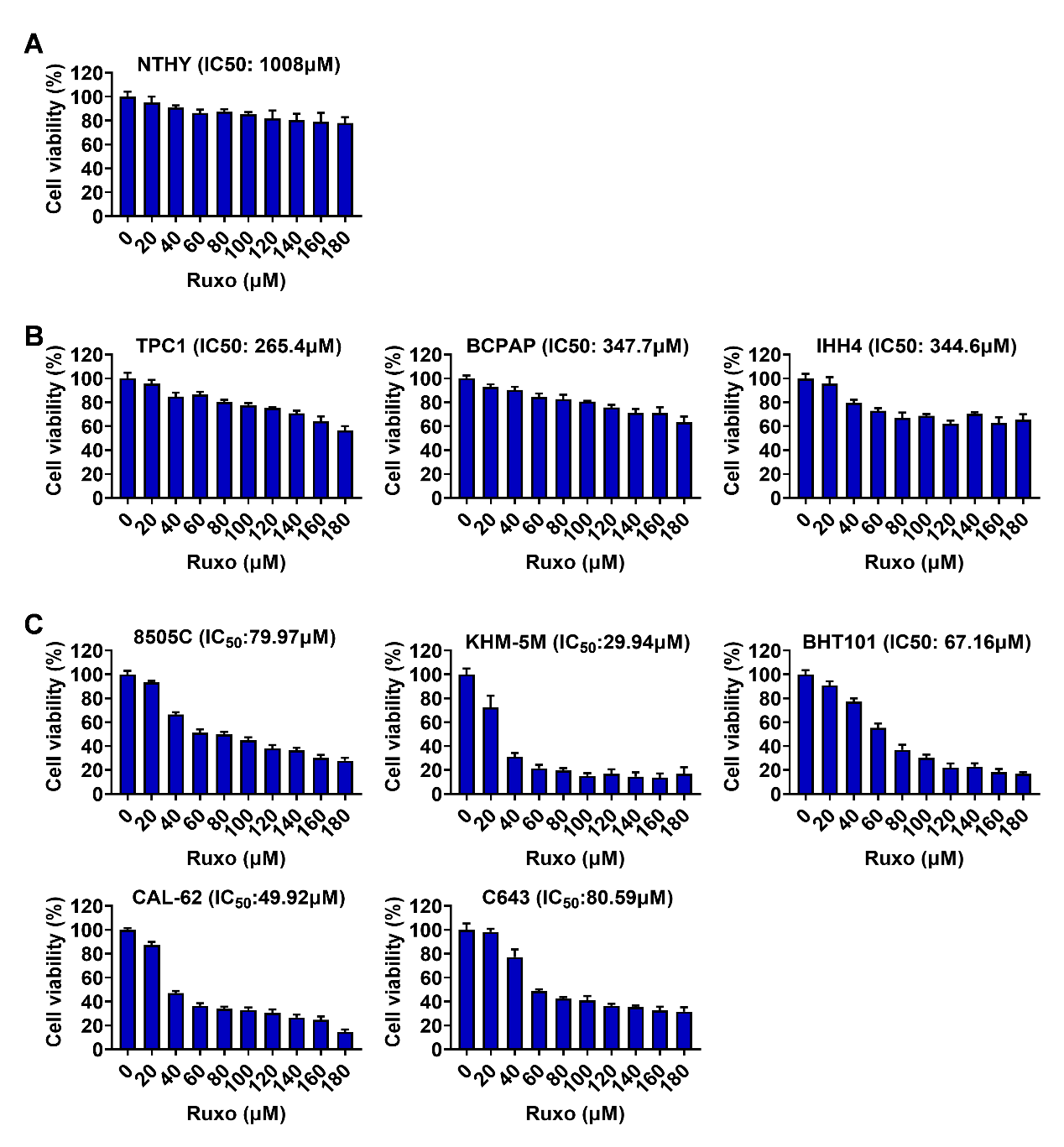


**Figure S2.** Ruxo impact on non-tumor thyroid cells and thyroid tumor cells. (A) CCK-8 assay was used to determined cell viability of different concentrations of Ruxo (0-180 μM) to normal thyroid epithelial cells NTHY (A); papillary thyroid cancer cells: TPC-1, BCPAP, IHH4 (B); ATC cells: 8505C, KHM-5M, BHT101, CAL62, C643 (C) after 24h. IC50 values were calculated from non-linear regression plots using the GraphPad. Values are presented as mean ± SD for n = 3


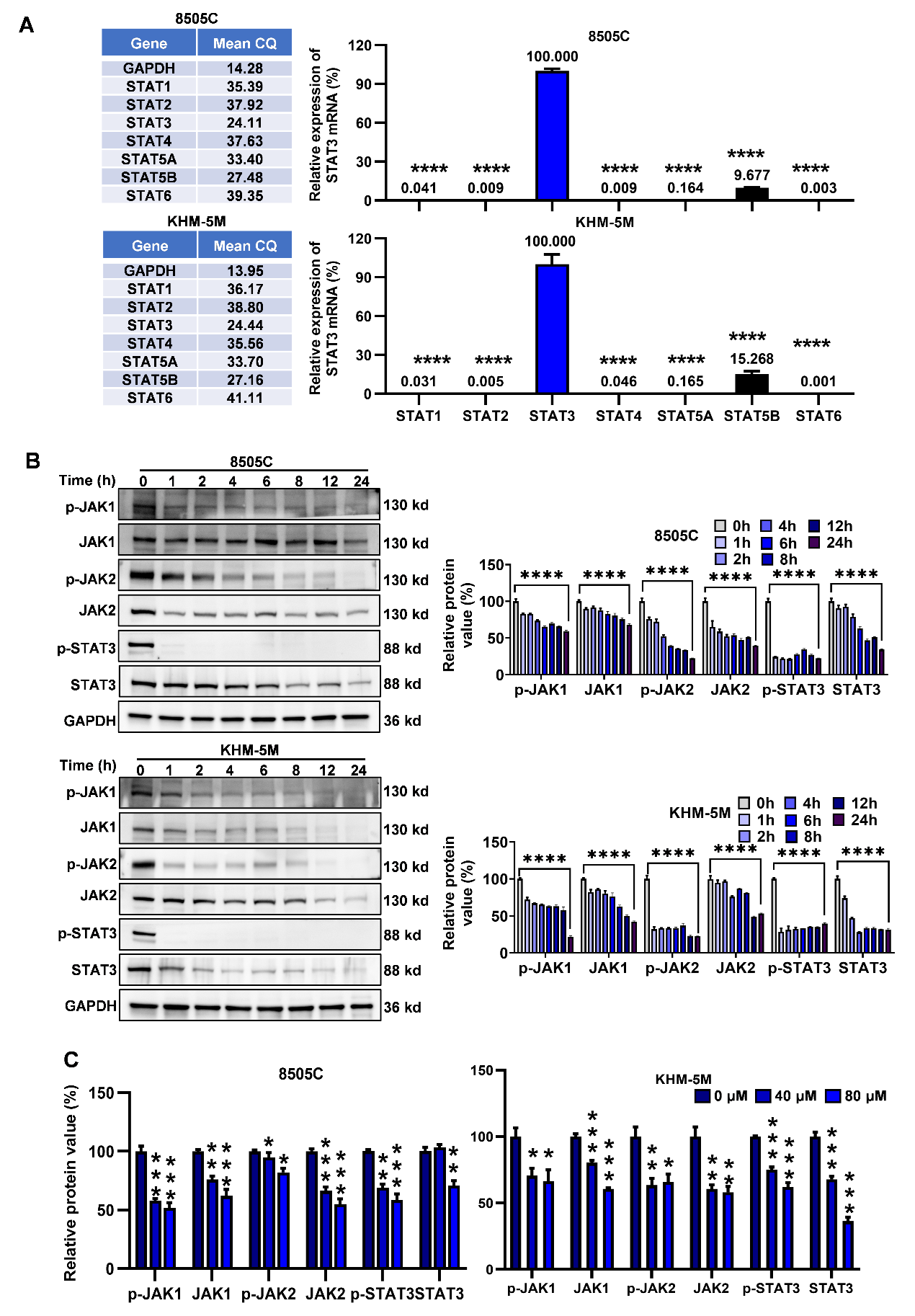


**Figure S3.** Ruxo inhibited JAK1/2-STAT3 pathway in ATC cells. (A) CQ values of STAT family protein and expression of STAT family proteins relative to STAT3 in ATC cells. (B) Quantitative and qualitative analyze of protein levels of JAK1/2 and STAT3 prototype and phosphorylated by extracting the proteins at various time intervals (0, 1 h, 2 h, 4 h, 6 h, 8 h, 12 h, 24 h) after administered Ruxo at a concentration of 80 μM to 8505C and KHM-5M cells. (C) Quantitative analyze of protein levels from JAK1/2-STAT3 pathway by western blot assays of Figure 2B. Values are presented as mean ± SD for n = 3, analyzed by one-way ANOVA using the Holm-Sidak method. **p* < 0. 05, ***p* < 0. 01, ****p* < 0. 001, *****p* < 0. 0001.


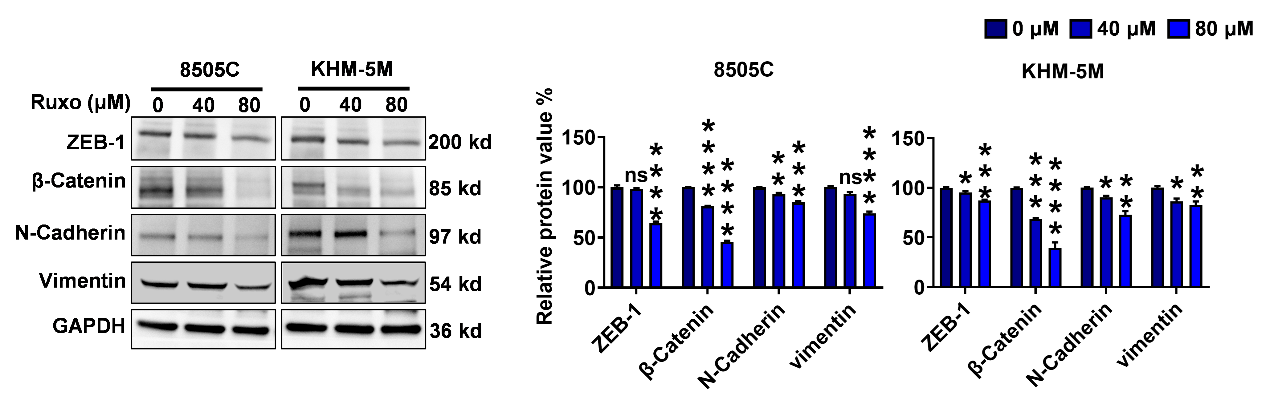


**Figure S4.** Effect of Ruxo on EMT pathway proteins in ATC cells. Quantitative and qualitative analyze of protein levels from EMT pathway in 8505C and KHM-5M cells after Ruxo (0, 40 and 80 μM) treatment for 24 h by western blot assays. Values are presented as mean ± SD for n = 3, analyzed by one-way ANOVA using the Holm-Sidak method. **p* < 0. 05, ***p* < 0. 01, ****p* < 0. 001, *****p* < 0. 0001.


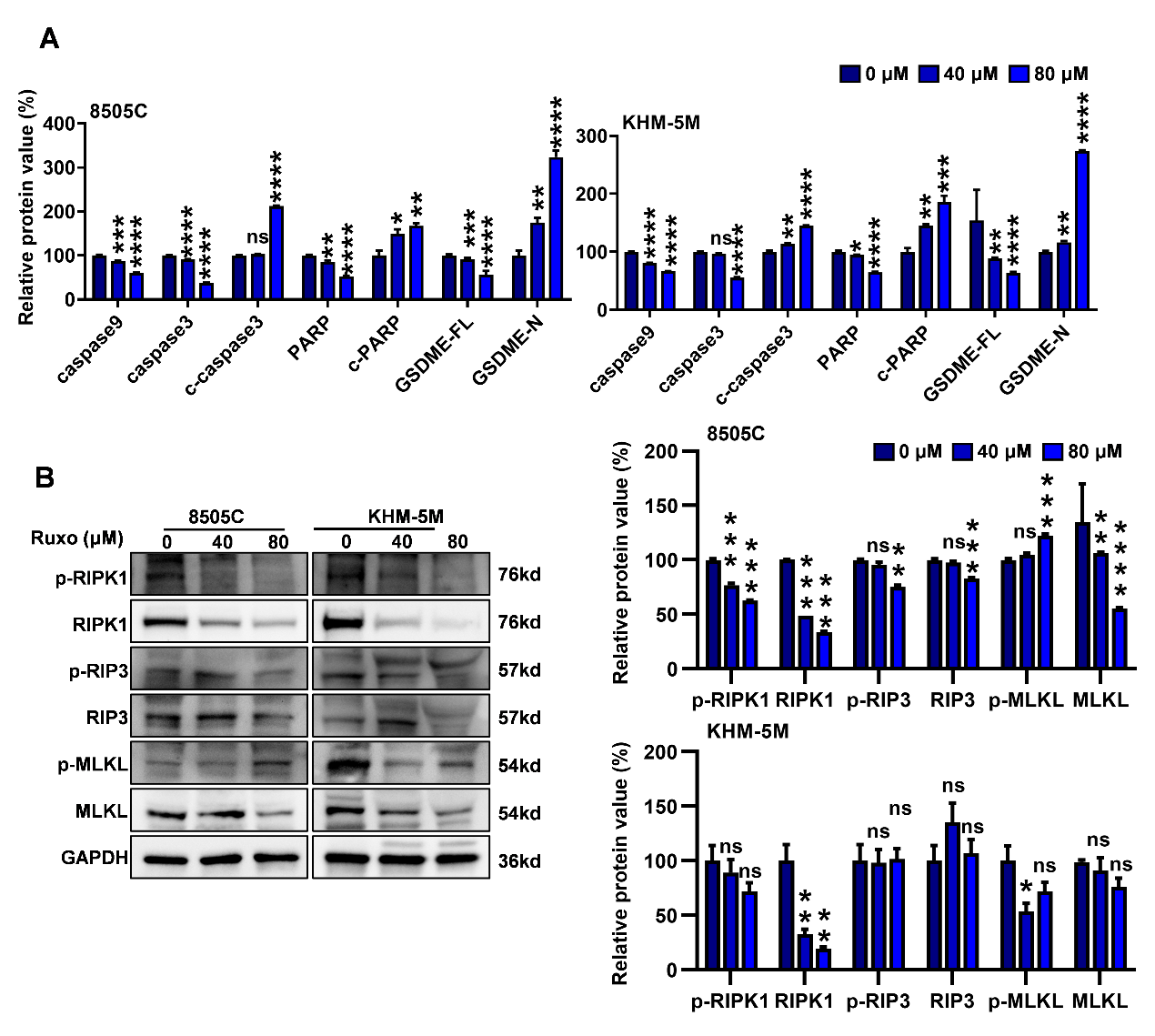


**Figure S5.** Ruxo induces apoptosis and pyroptosis in ATC cells. (A) Quantitative analyze of caspase 9, caspase 3, c-caspase 3, PARP, c-PARP, full-length GSDME and GSDME-N terminus protein levels in 8505C and KHM-5M cells after Ruxo (0, 40 and 80 μM) treatment for 24 h by western blot. (B) Quantitative and qualitative analyze of necroptosis markers protein levels in 8505C and KHM-5M cells after Ruxo (0, 40 and 80 μM) treatment for 24 h by western blot assays. Values are presented as mean ± SD for n = 3, analyzed by one-way ANOVA using the Holm-Sidak method. **p* < 0. 05, ***p* < 0. 01, ****p* < 0. 001, *****p* < 0. 0001.


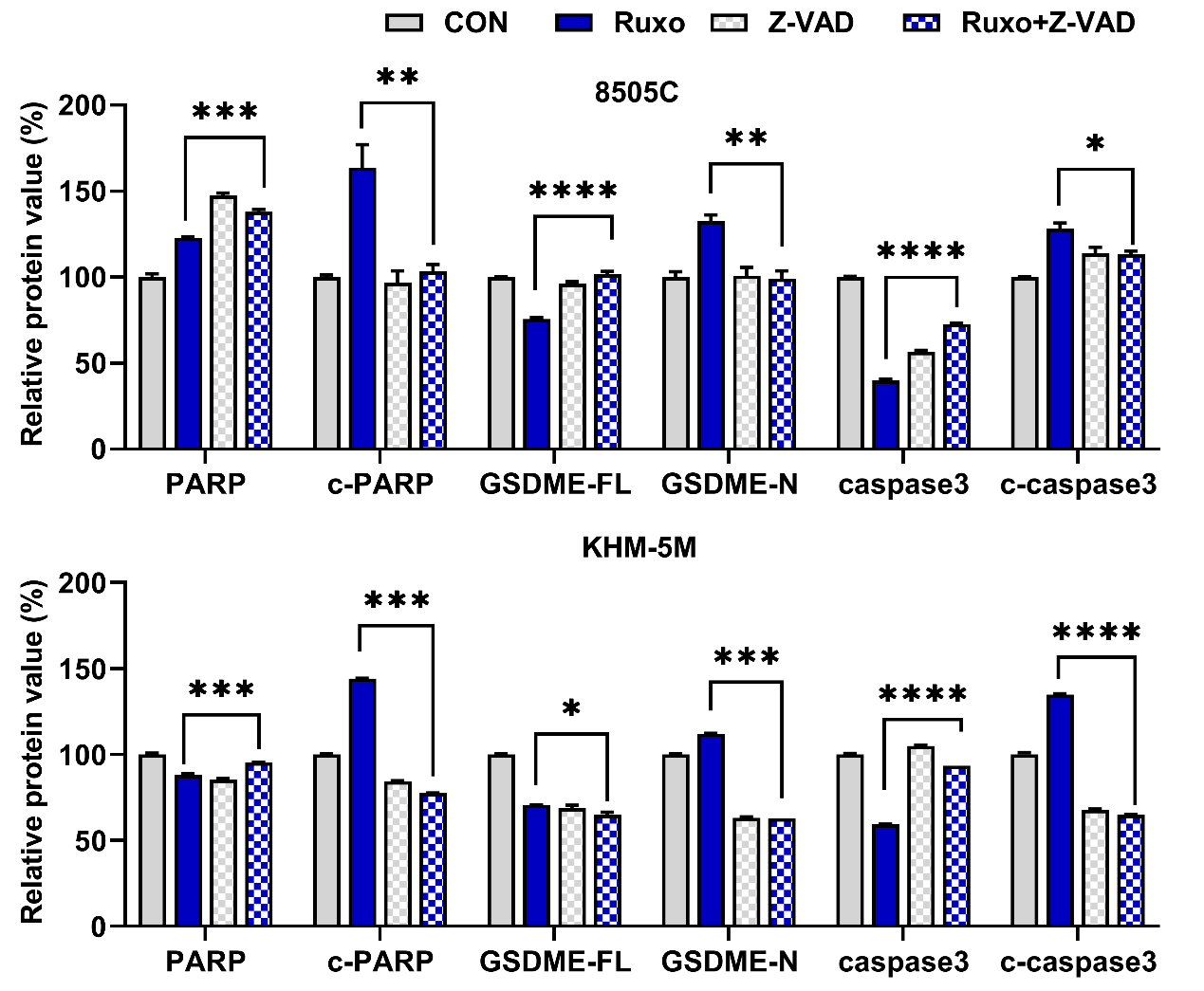


**Figure S6.** Ruxo induces Caspase3/GSDME-dependent pyroptosis in ATC cells. Quantitative analyze of caspase 3, c-caspase 3, PARP, c-PARP, full-length GSDME and GSDME-N terminus protein levels in 8505C and KHM-5M cells after Ruxo (0, 40 μM) treatment for 24 h, with or without Z-VAD by western blot. Values are presented as mean ± SD for n = 3, analyzed by two-way ANOVA using the Tukey method. **p* < 0. 05, ***p* < 0. 01, ****p* < 0. 001, *****p* < 0. 0001.


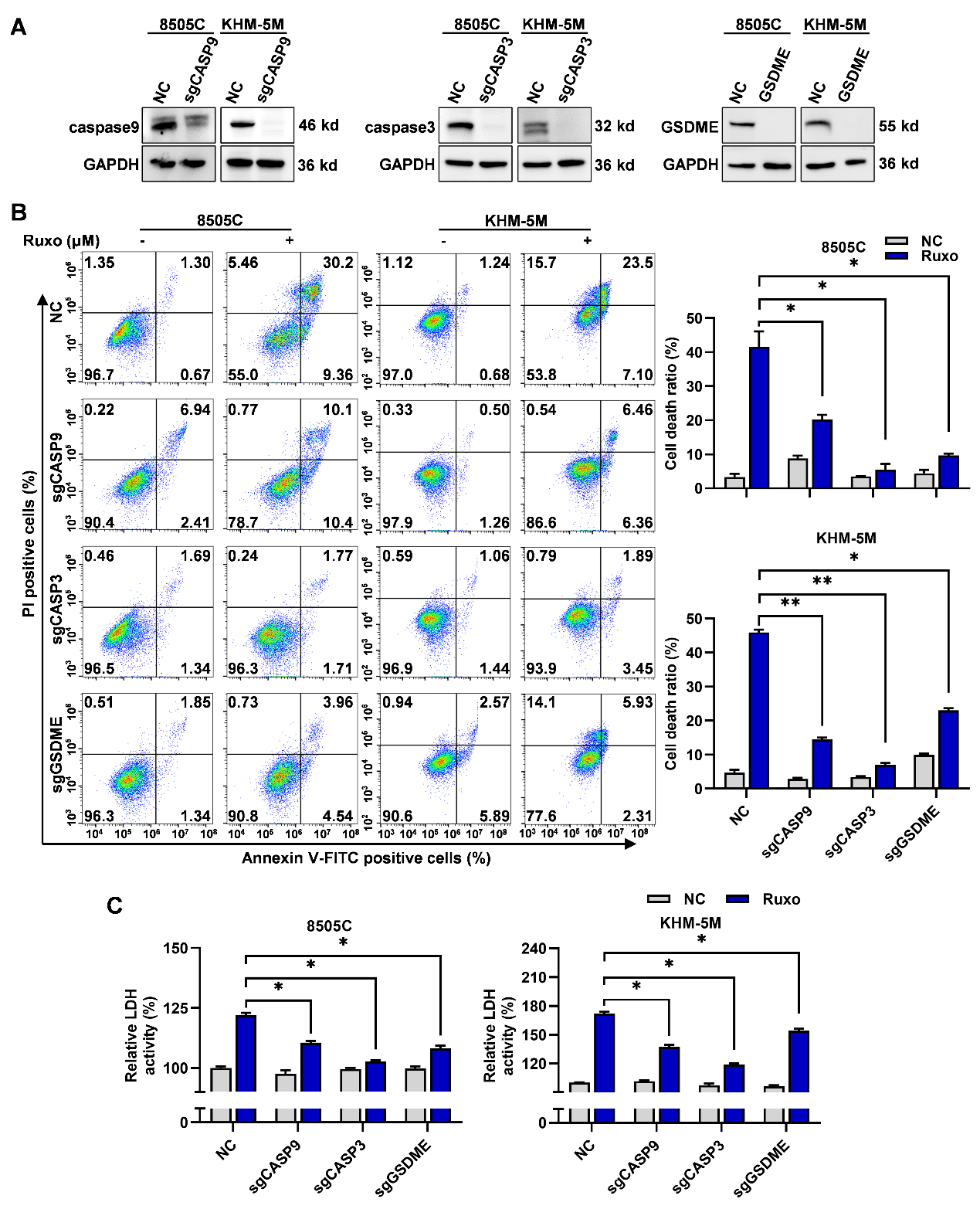


**Figure S7.** Ruxo induces Caspase3/GSDME-dependent pyroptosis in ATC cells. (A) Stable knockdown of caspase3 and GSDME in ATC cells using CRISPR/Cas9 technology. Western blot was used to validate knockdown efficiency. (B) 8505C wild-type control, sgCASP9, sgCASP3 and sgGSDME cells were treated with Ruxo (80Μm) for 36 h; KHM-5M wild-type control, sgCASP9, sgCASP3 and sgGSDME cells were treated with Ruxo (80μm) for 18 h before performing flow cytometry to measured cell deaths of ATC cells. (C) Relative LDH activities in the lysate of cells were measured by the LDH assay Kit. Values are presented as mean ± SD for n = 3, analyzed by two-way ANOVA using the Tukey method. **p* < 0. 05, ***p* < 0. 01, ****p* < 0. 001, *****p* < 0. 0001.


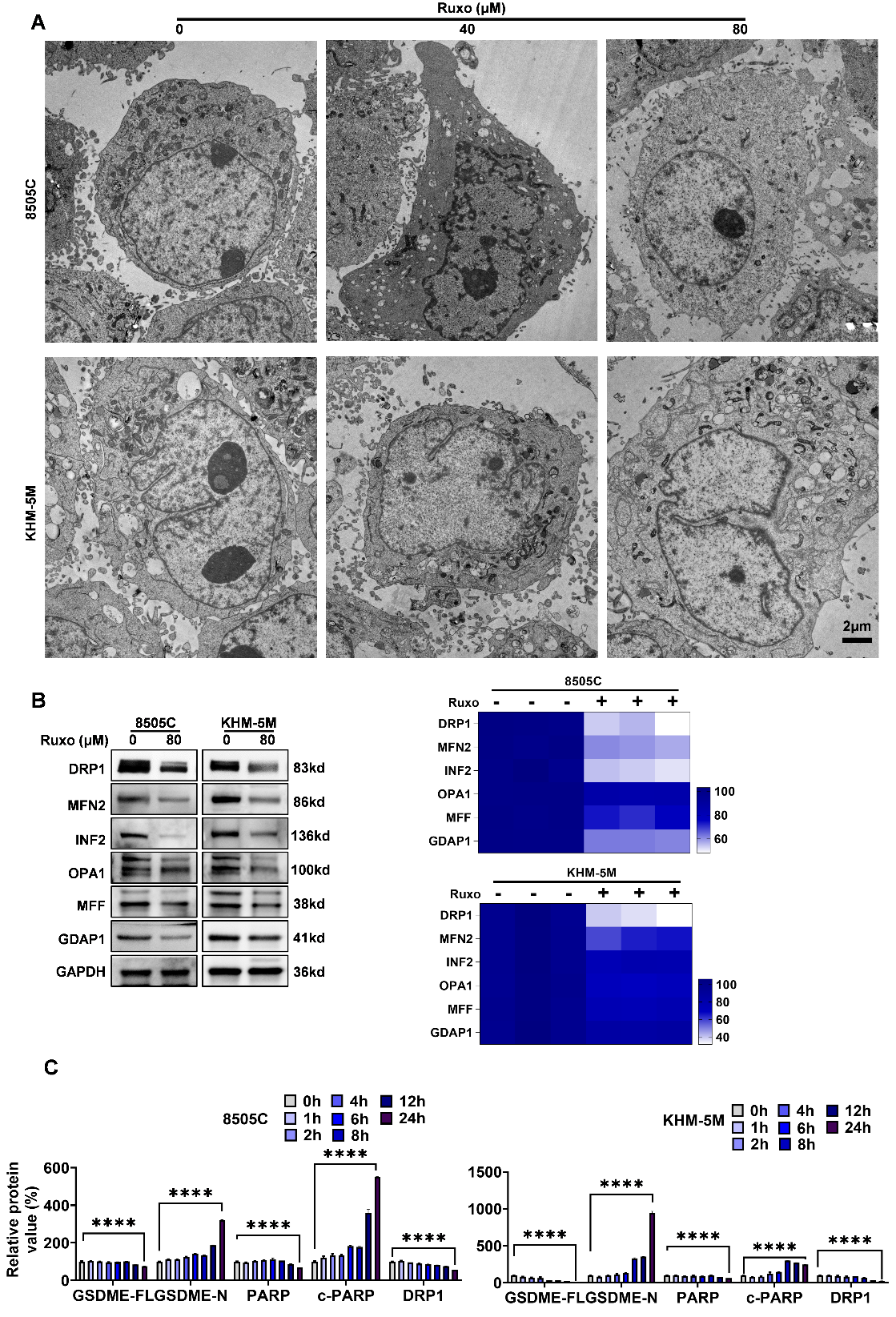


**Figure S8.** Ruxo induces mitochondrial fission disorder in ATC cell. (A) Transmission electron microscopy was used to observe mitochondrial morphological changes in 8505C and KHM-5M cells treated with Ruxo (0, 40, 80 μM for 8 h). (B) Quantitative and qualitative analyze by western blot assays of levels of mitochondrial fission-related genes, after treating with Ruxo (0, and 80 μM) for 24 h. (C) Quantitative analyze of DRP1, PARP, c-PARP, full-length GSDME and GSDME-N terminus protein levels in 8505C and KHM-5M cells after Ruxo (0, 80 μM) treatment for various times intervals (0, 1 h, 2 h, 4 h, 6 h, 8 h, 12 h, 24 h). Values are presented as mean ± SD for n = 3, analyzed by one-way ANOVA using the Holm-Sidak method, or two-way ANOVA using the Tukey method for multiple comparisons. **p* < 0. 05, ***p* < 0. 01, ****p* < 0. 001, *****p* < 0. 0001.


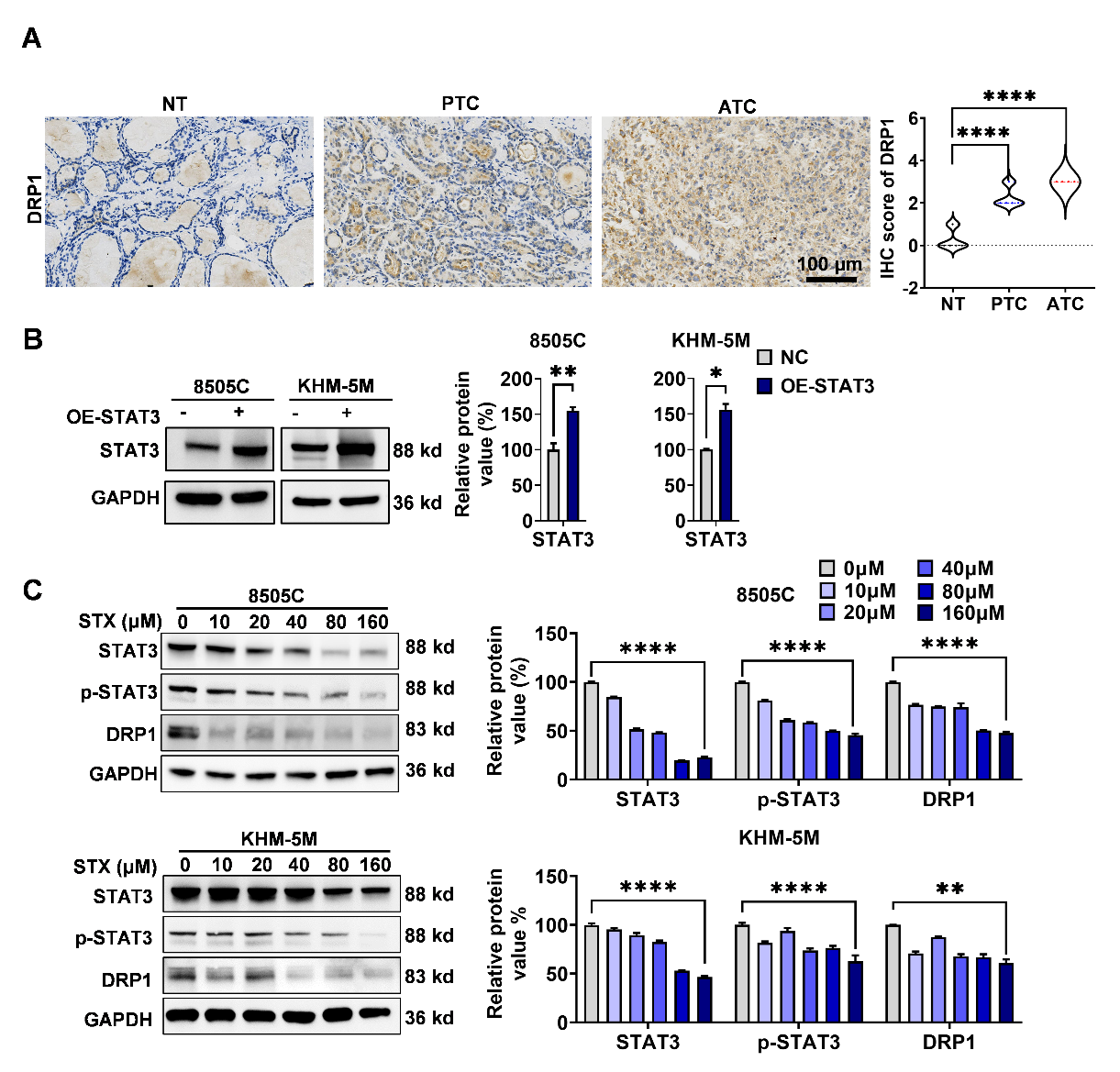


**Figure S9.** Ruxo downregulates DRP1 through inhibition of JAK1/2-STAT3 signaling pathway activation. (A) IHC was used to determine the expression of DRP1 of tissue samples from our center. (B) STAT3 were analyzed by western blot after transfection of overexpression plasmid of STAT3 for 48 h. (C) Quantitative and qualitative analyze of STAT3, p-STAT3, DRP1 and GAPDH protein levels in 8505C and KHM-5M cells after treated with STX-0119 (STAT3 inhibitor) (0, 10, 20, 40, 80, 160μM for 24 h). Values are presented as mean ± SD for n = 3, analyzed by one-way ANOVA using the Holm-Sidak method, or two-way ANOVA using the Tukey method for multiple comparisons. **p* < 0. 05, ***p* < 0. 01, ****p* < 0. 001, *****p* < 0. 0001.


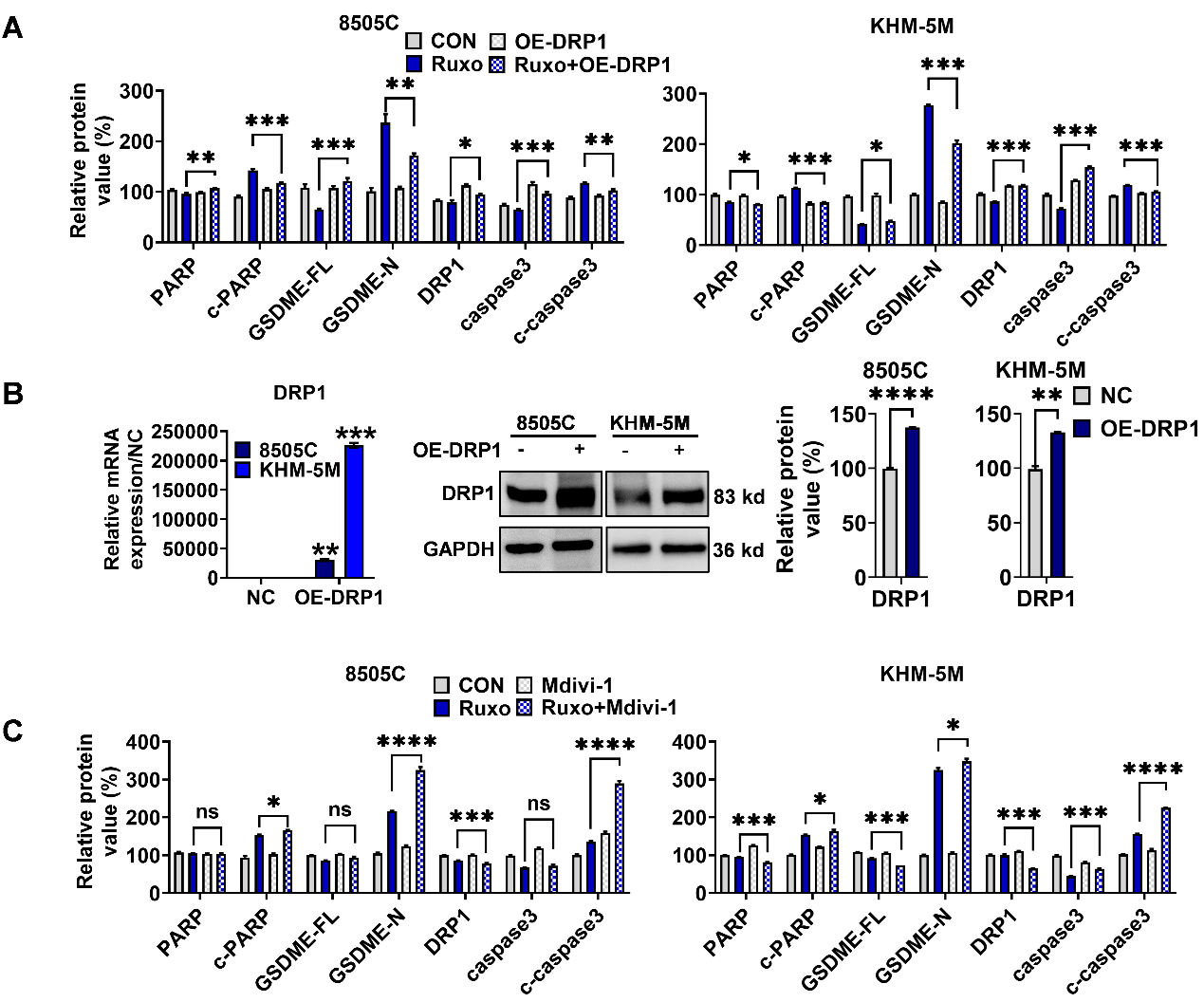


**Figure S10.** DRP1 expression is closely associated with Ruxo-induced ATC cell death. (A) quantitative analyze of DRP1, caspase 3, c-caspase 3, PARP, c-PARP, full-length GSDME and GSDME-N terminus protein levels of Ruxo -treated ATC cells (8505C, 80 μM and KHM-5M, 20 μM, for 24 h) with or without transfection of DRP1 overexpression plasmid. (B) DRP1 were analyzed by western blot after transfection of DRP1 overexpression plasmid. (C) quantitative analyze of DRP1, caspase 3, c-caspase 3, PARP, c-PARP, full-length GSDME and GSDME-N terminus protein levels of Ruxo -treated ATC cells (8505C, 40 μM and KHM-5M, 20 μM, for 24 h) with or without Mdivi-1(a DRP1 inhibitor). Values are presented as mean ± SD for n = 3, analyzed by one-way ANOVA using the Holm-Sidak method. **p* < 0. 05, ***p* < 0. 01, ****p* < 0. 001, *****p* < 0. 0001.


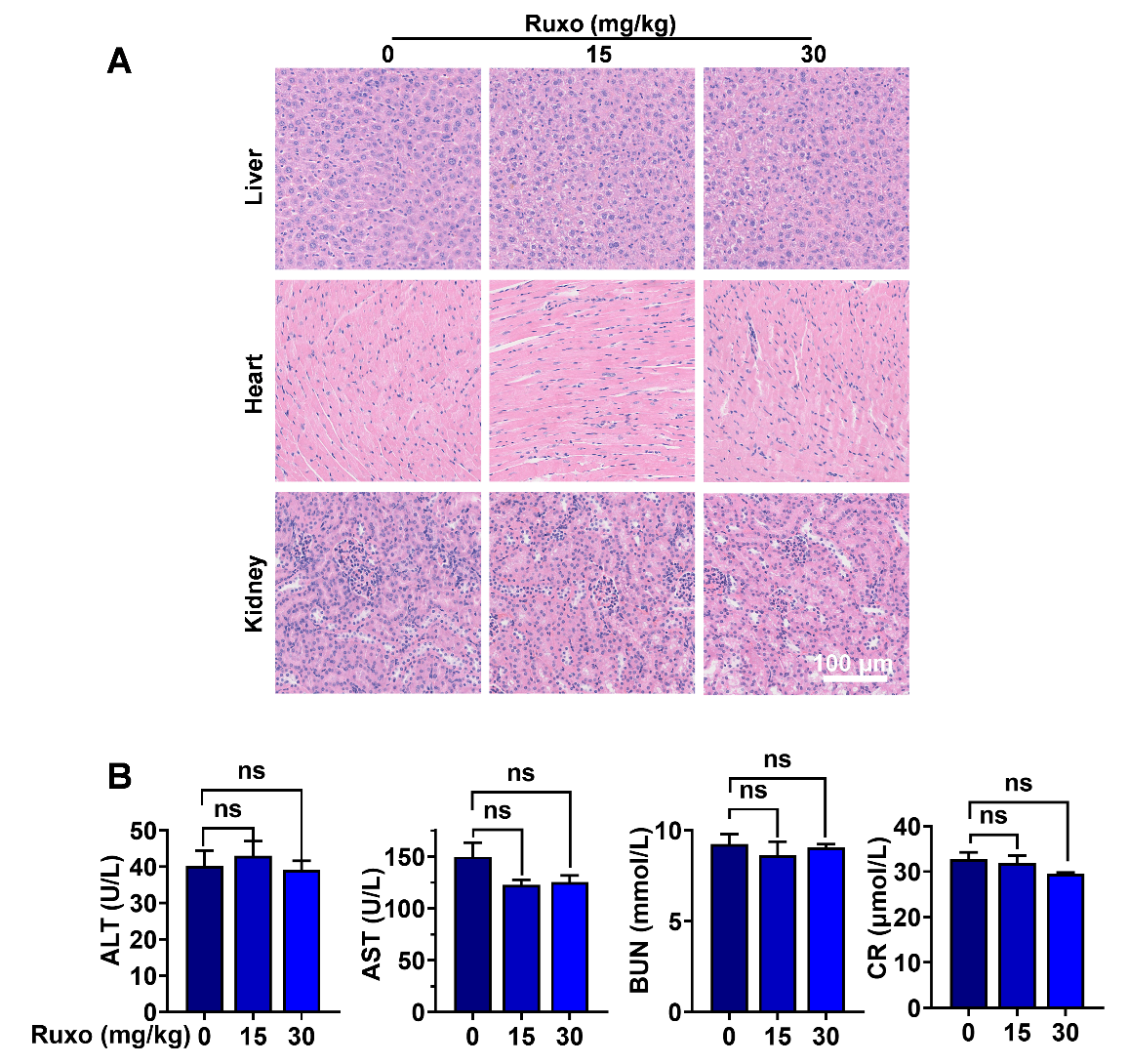


**Figure S11.** Ruxo showed no toxicity *in vivo*. (A) Liver, heart and kidney HE staining of each group. (B) Quantitative chart about ALT, AST, BUN and CR of each group. Values are presented as mean ± SD for n = 3, analyzed by one-way ANOVA using the Holm-Sidak method (B). **p* < 0. 05, ***p* < 0. 01.
