# Supplementary material for: Ruxolitinib induces apoptosis and pyroptosis of anaplastic thyroid cancer via the transcriptional inhibition of DRP1-mediated mitochondrial fission
Source: Cell Death Dis. 2024 Feb 9;15(2):125. doi: 10.1038/s41419-024-06511-1 (PMC10858168; doi:10.1038/s41419-024-06511-1)
Supplement: Supplementary file 3 — supplement table [file 41419_2024_6511_MOESM3_ESM.docx]

Table 1. The PCR primer sequences for genes

| **Gene** | **Forward/Reverse** | **Sequences (5′-3′)** |
| --- | --- | --- |
| STAT3 | Forward | ATCACGCCTTCTACAGACTGC |
|  | Reverse | CATCCTGGAGATTCTCTACCACT |
| DRP1 | Forward | TCACCCGGAGACCTCTCATTC |
|  | Reverse | GGTTCAGGGCTTACTCCCTTAT |
| INF2 | Forward | GCGTTGCACGTATCTCCGA |
|  | Reverse | CACCATCACGTTGGATGTGTC |
| MFF | Forward | ACTGAAGGCATTAGTCAGCGA |
|  | Reverse | TCCTGCTACAACAATCCTCTCC |
| MFN2 | Forward | CTCTCGATGCAACTCTATCGTC |
|  | Reverse | TCCTGTACGTGTCTTCAAGGAA |
| OPA1 | Forward | TGTGAGGTCTGCCAGTCTTTA |
|  | Reverse | TGTCCTTAATTGGGGTCGTTG |
| GDAP1 | Forward | ATGCGTTTGAACTCAACTGGA |
|  | Reverse | TCAGGCATTAACCTGGGTGTT |
| ACTB | Forward | CATGTACGTTGCTATCCAGGC |
|  | Reverse | CTCCTTAATGTCACGCACGAT |

Table 2. The primary antibodies for Western blotting

| **Antibody** | **Company** | **Number** |
| --- | --- | --- |
| PARP | Cell Signaling Technology | 9532 |
| Caspase 9 | Cell Signaling Technology | 9502 |
| Bax | Cell Signaling Technology | 5023 |
| RIP3 | Cell Signaling Technology | 10188T |
| p-RIP3 | Cell Signaling Technology | 93654T |
| MLKL | Cell Signaling Technology | 14993T |
| p-MLKL | Cell Signaling Technology | 91689T |
| GAPDH | Proteintech | 66004-1-Ig |
| Bcl-2 | Proteintech | 60178-1-Ig |
| DRP1 | Proteintech | 12957-1-AP |
| RIPK1 | Proteintech | 17519-1-AP |
| p-RIPK1 | Proteintech | 66854-1-Ig |
| MFN2 | Proteintech | 12186-1-AP |
| INF2 | Proteintech | 20466-1-AP |
| OPA1 | Proteintech | 27733-1-AP |
| MFF | Proteintech | 17090-1-AP |
| GDAP1 | Proteintech | 68083-1-Ig |
| ZEB1 | Proteintech | 21544-1-AP |
| β-Catenin | Proteintech | 66379-1-Ig |
| N-Cadherin | Proteintech | 66219-1-Ig |
| Vimentin | Proteintech | 10366-1-AP |
| STAT3 (phospho Y705) | Abcam | 76315 |
| JAK1 (phospho Y1034+Y1035) | Abcam | 138005 |
| GSDME | Abcam | 215191 |
| Caspase 3 | Abcam | 32351 |
| JAK1 | HuaBio | 1705-84 |
| JAK2 | HuaBio | 1607-35 |
| JAK2 (phosphor Y1007+Y1008) | HuaBio | 1607-34 |
| STAT3 | HuaBio | 1607-38 |
| cleaved-PARP | HuaBio | 1608-10 |
| active Caspase 3 | HuaBio | 1602-47 |

Table 3. Potential direct binding site for STAT3 in the promoter region of DRP1

| **Matrix ID** | **Name** | **Score** | **Relative score** | **Sequence ID** | **Start** | **End** | **Strand** | **Predicted sequence** |
| --- | --- | --- | --- | --- | --- | --- | --- | --- |
| MA0144.2 | STAT3 | 11.79117 | 0.9404 | DNM1L | 748 | 758 | + | TTGCCAGGAAT |
| MA0144.2 | STAT3 | 10.33875 | 0.922804 | DNM1L | 1315 | 1325 | + | CATCCTGGAAA |
