# Supplementary figures and images for: Ruxolitinib induces apoptosis and pyroptosis of anaplastic thyroid cancer via the transcriptional inhibition of DRP1-mediated mitochondrial fission
Source: Cell Death Dis. 2024 Feb 9;15(2):125. doi: 10.1038/s41419-024-06511-1 (PMC10858168; doi:10.1038/s41419-024-06511-1)

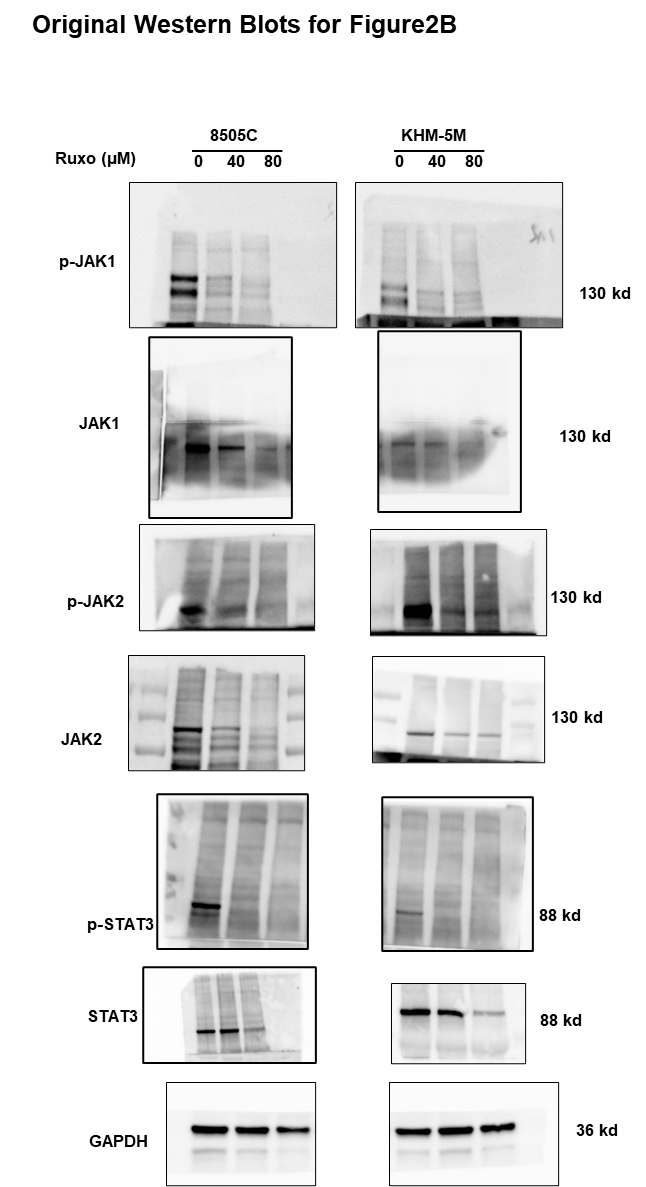


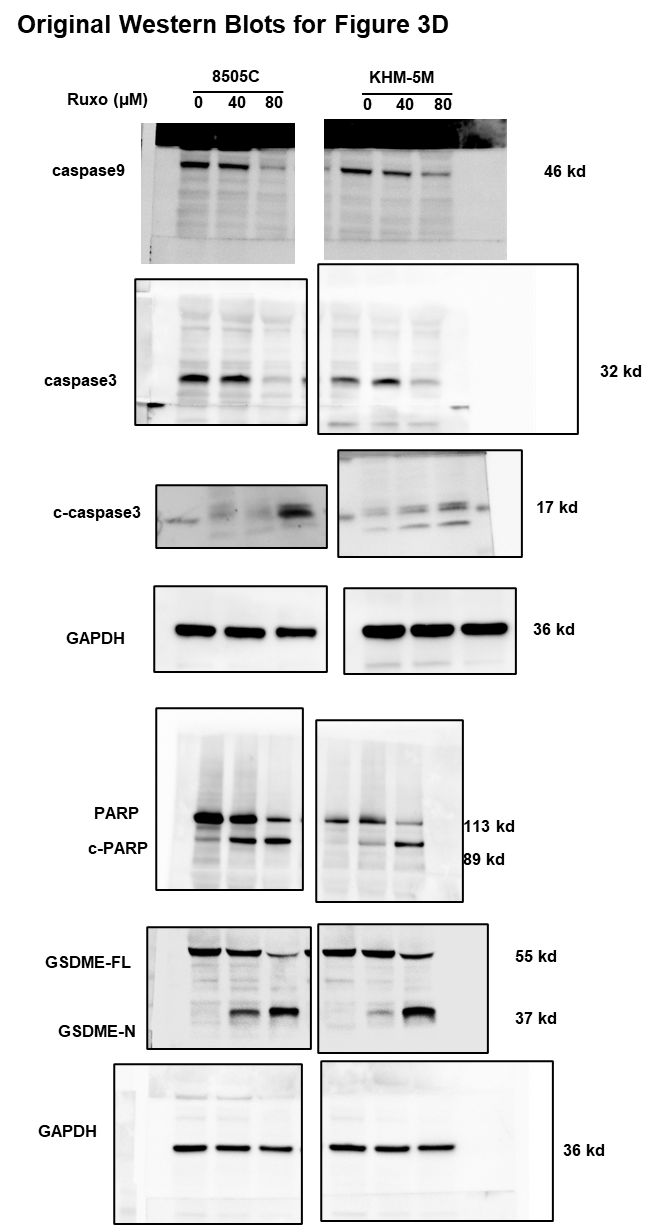


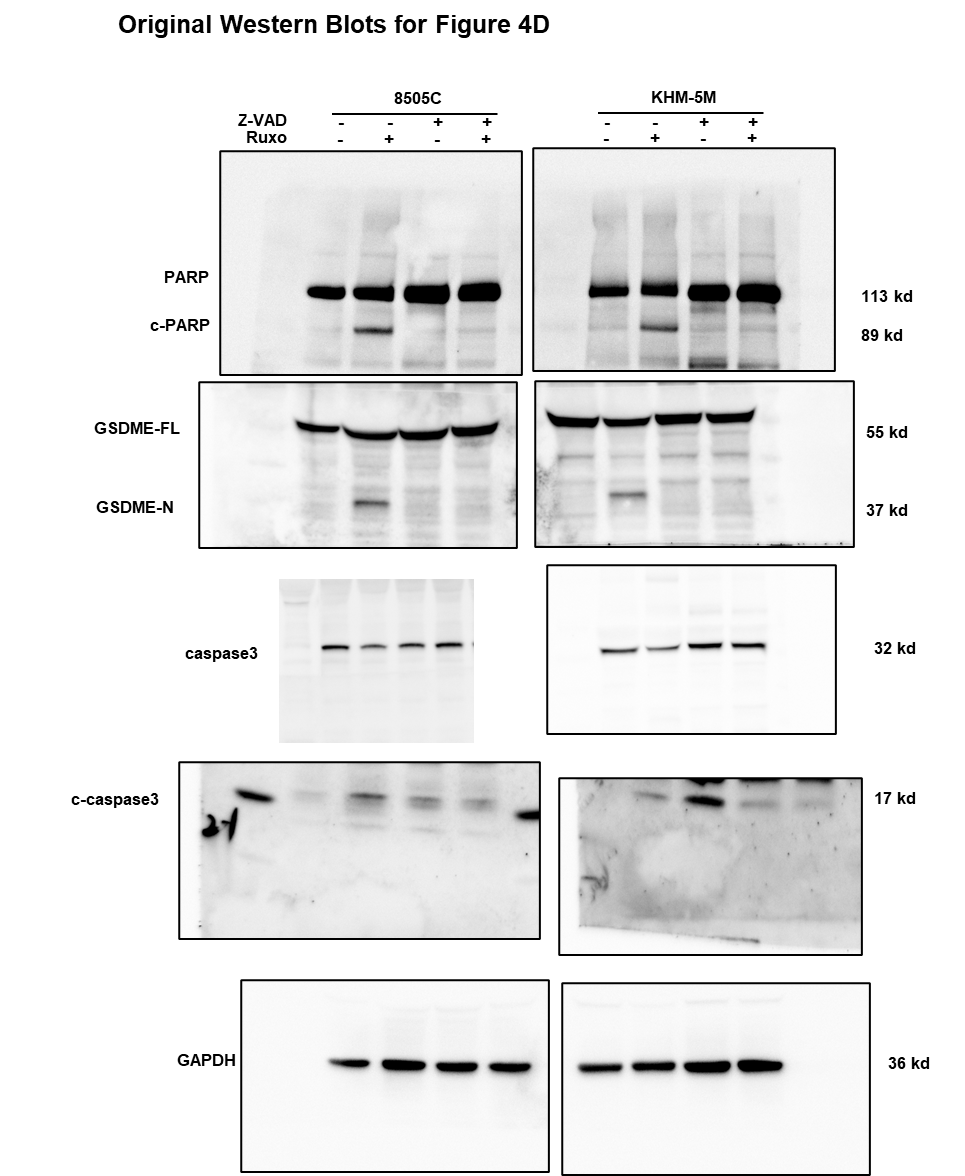


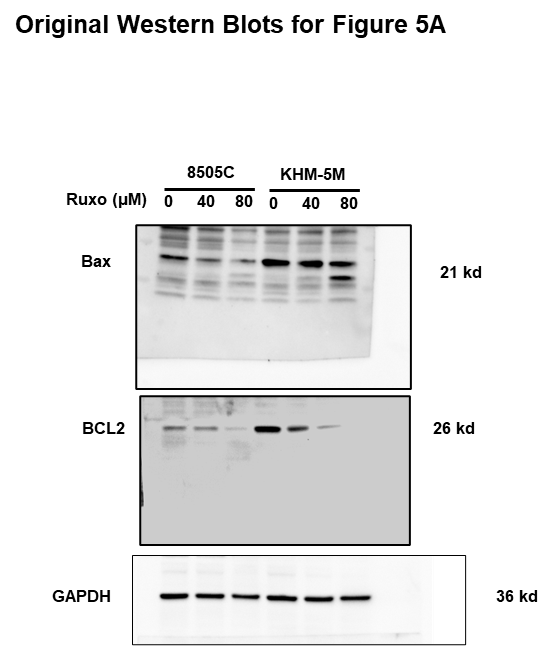


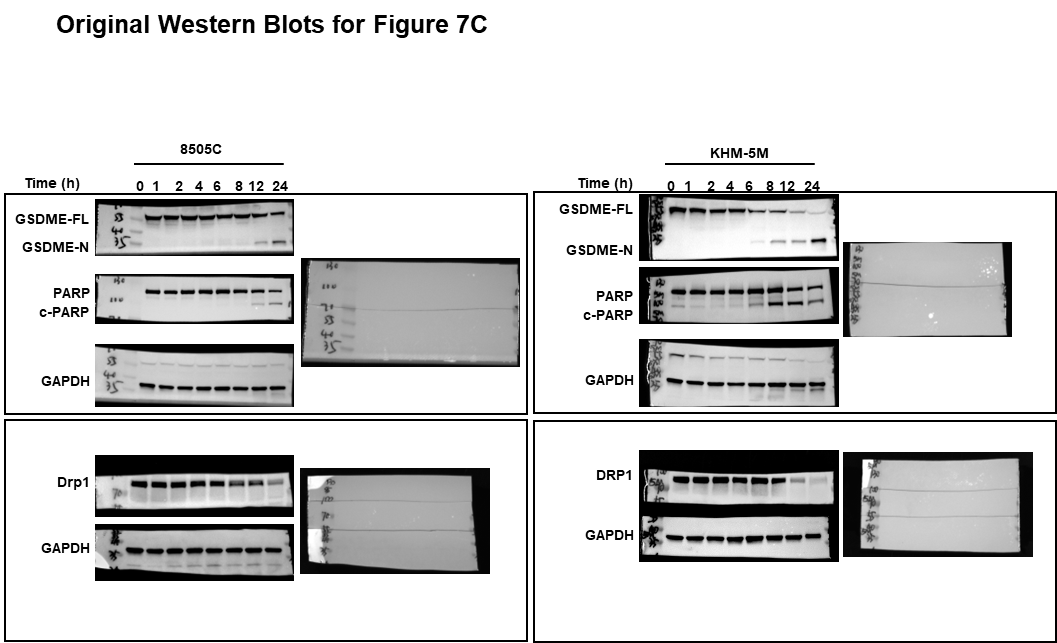


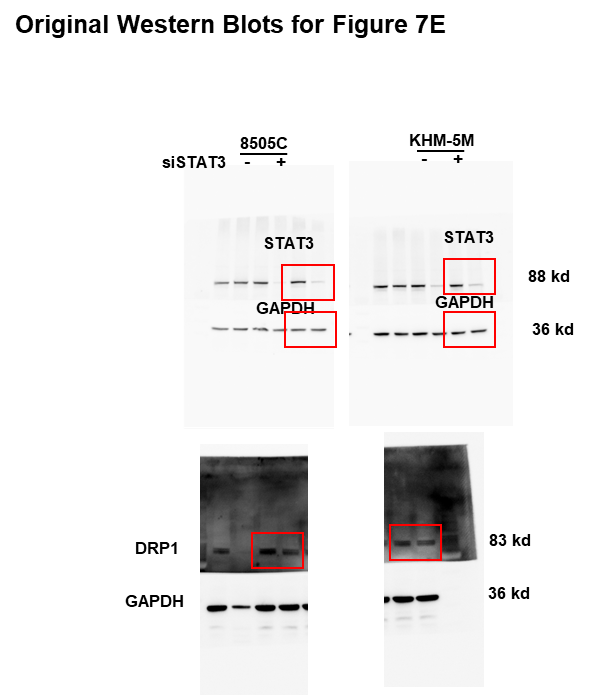


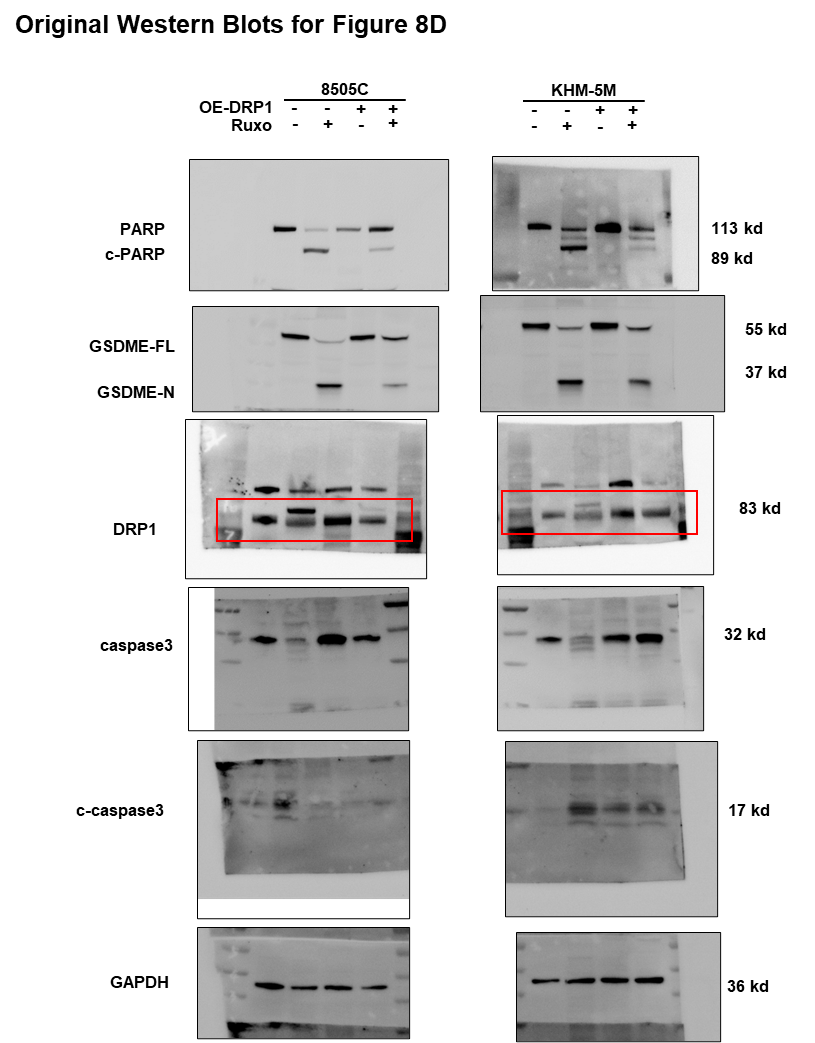


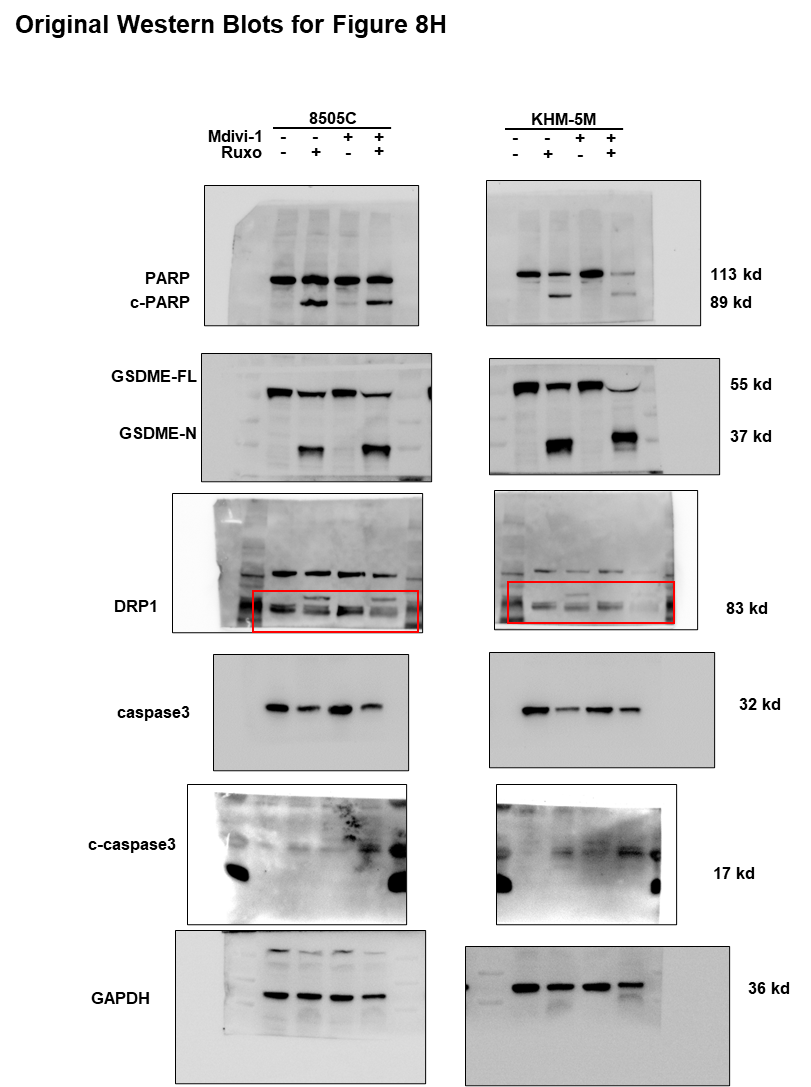


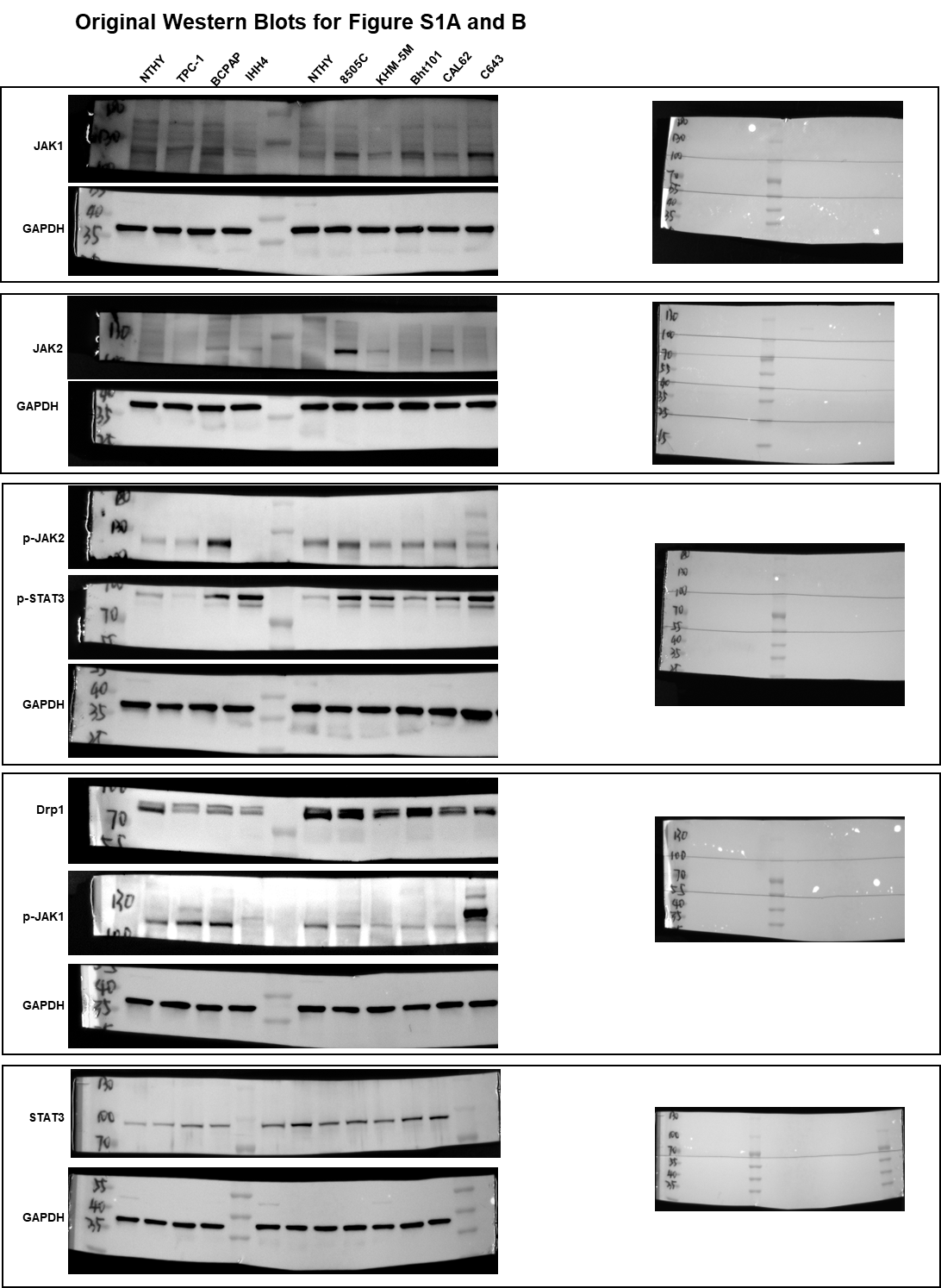


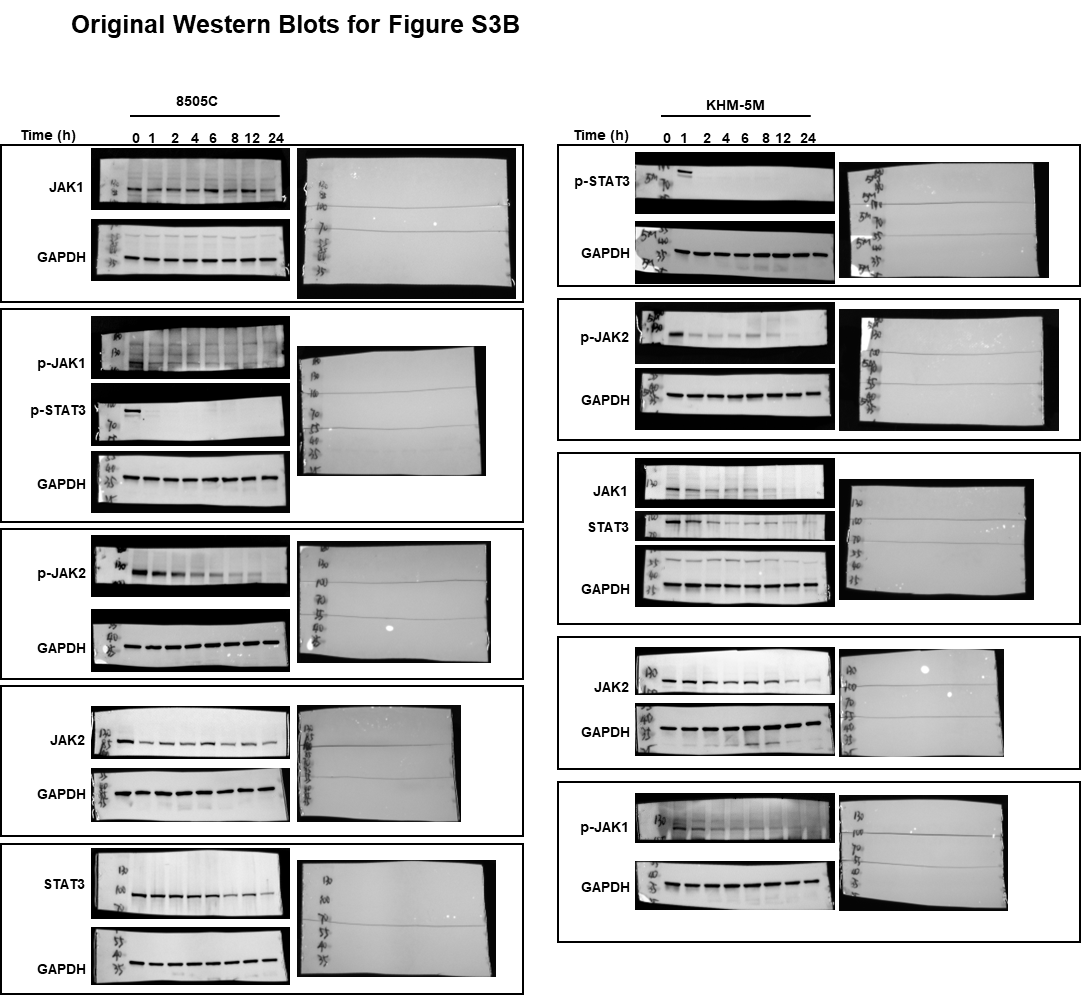


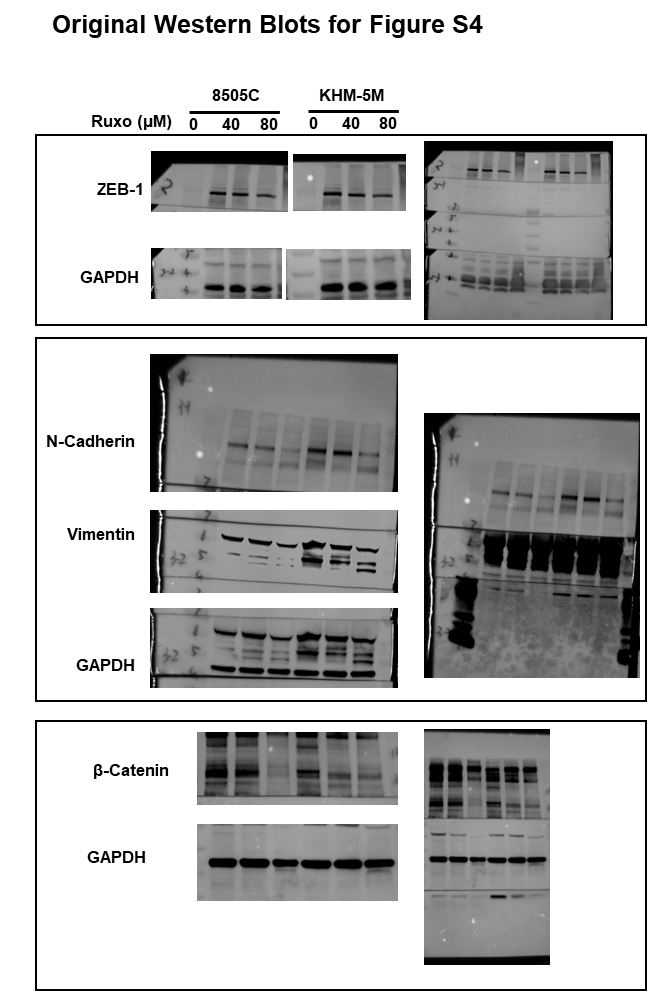


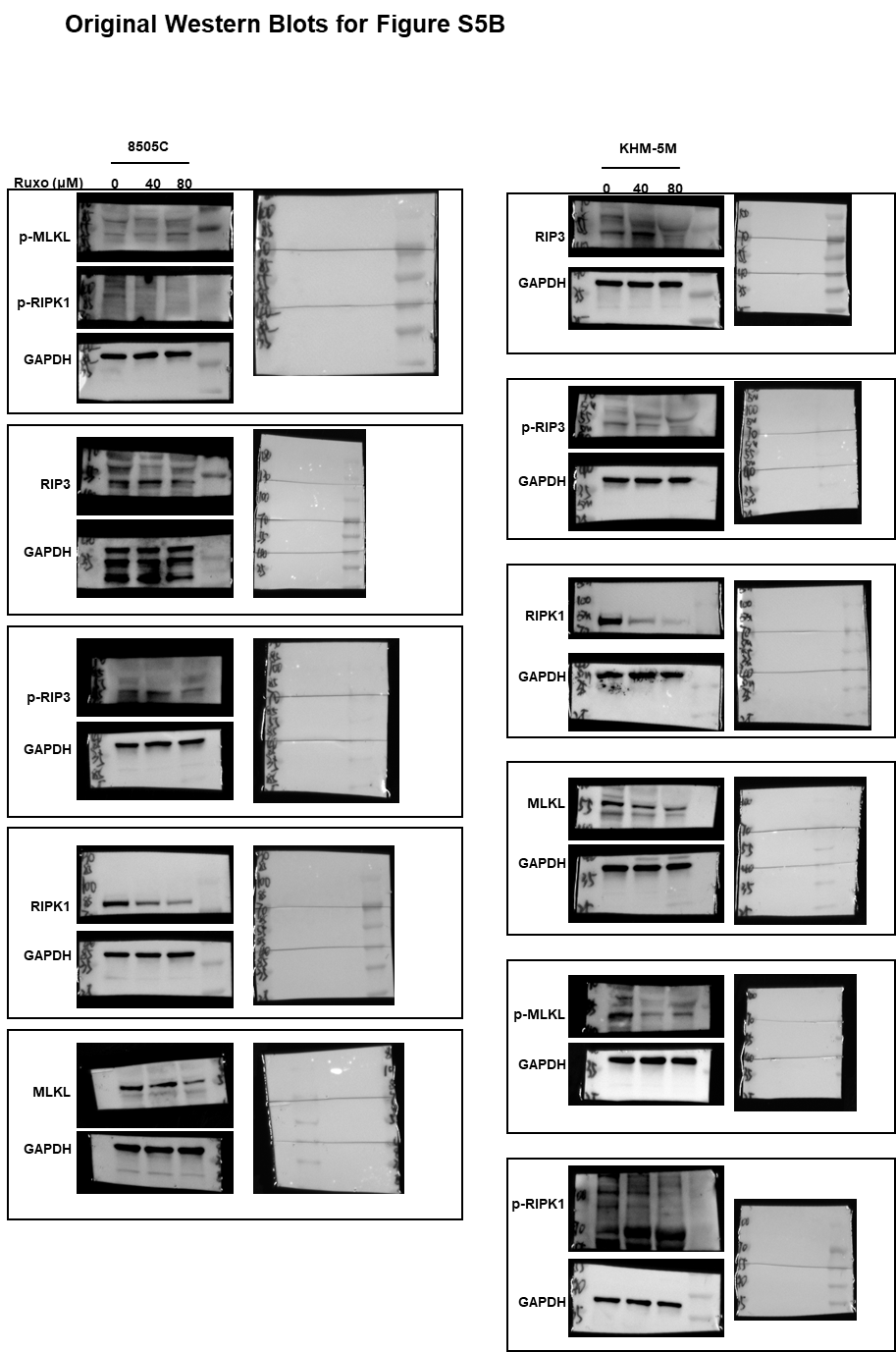


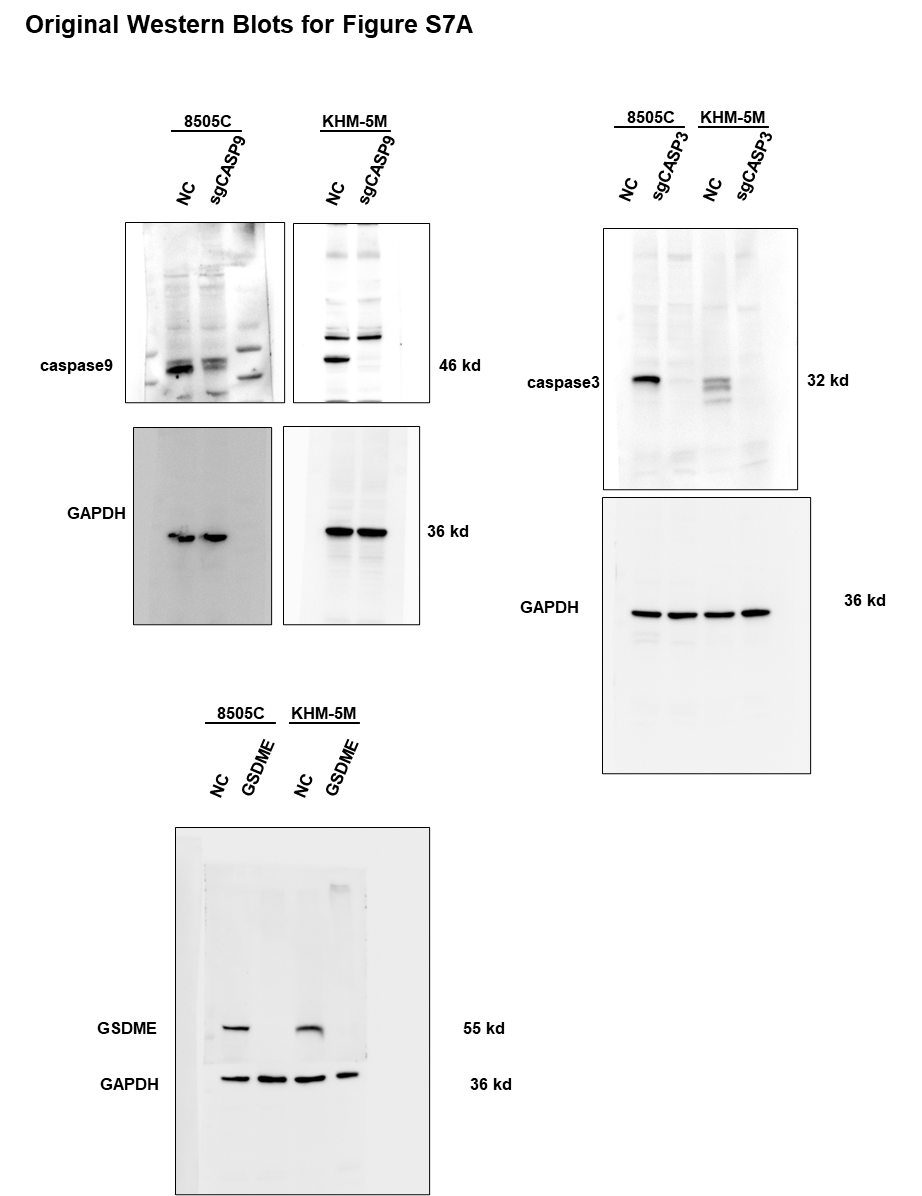


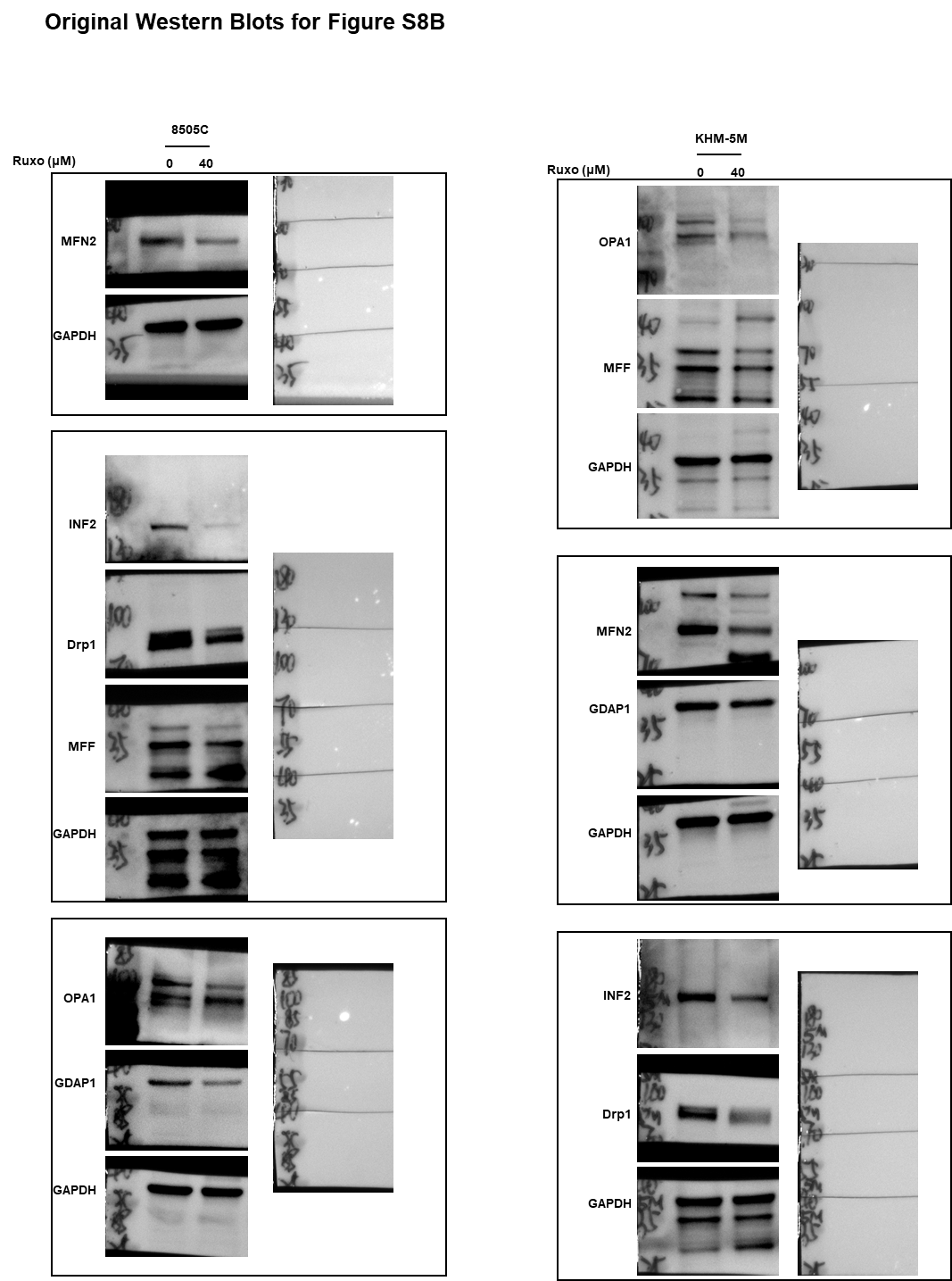


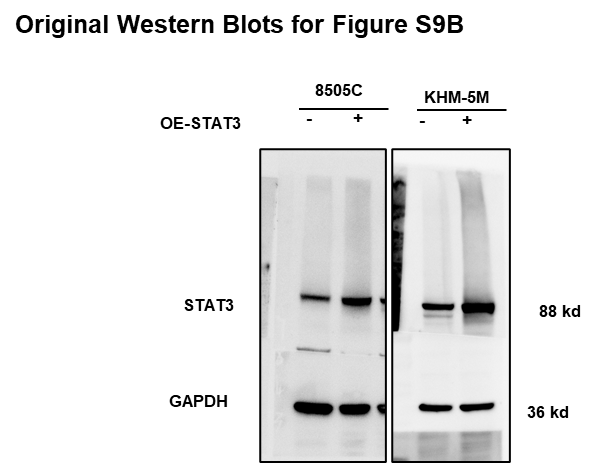


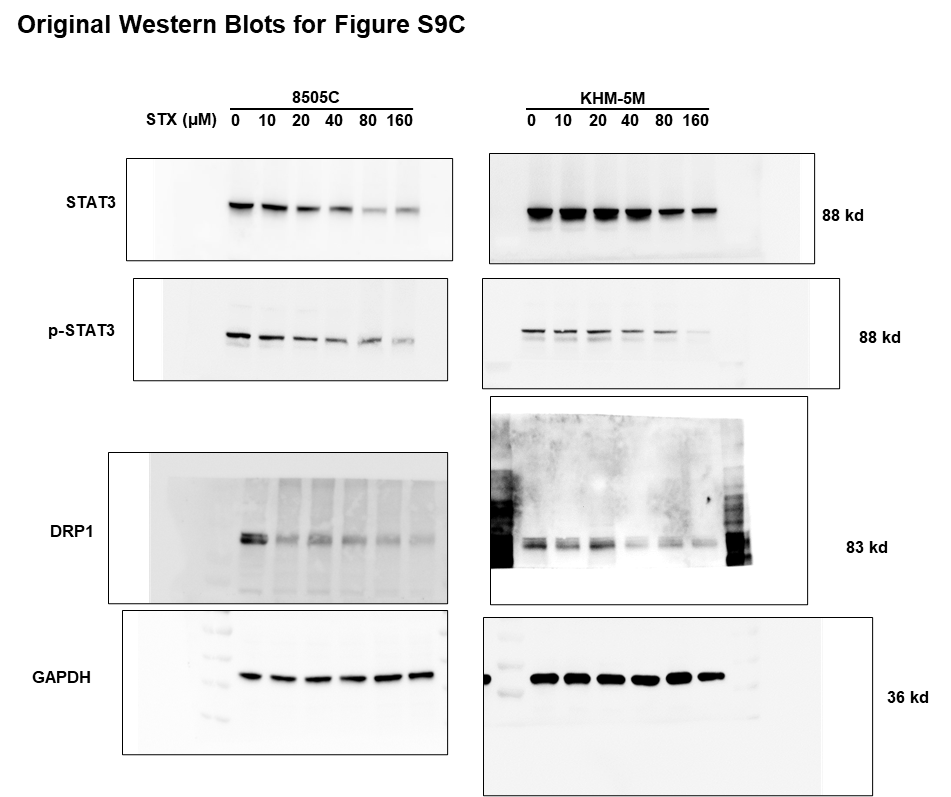


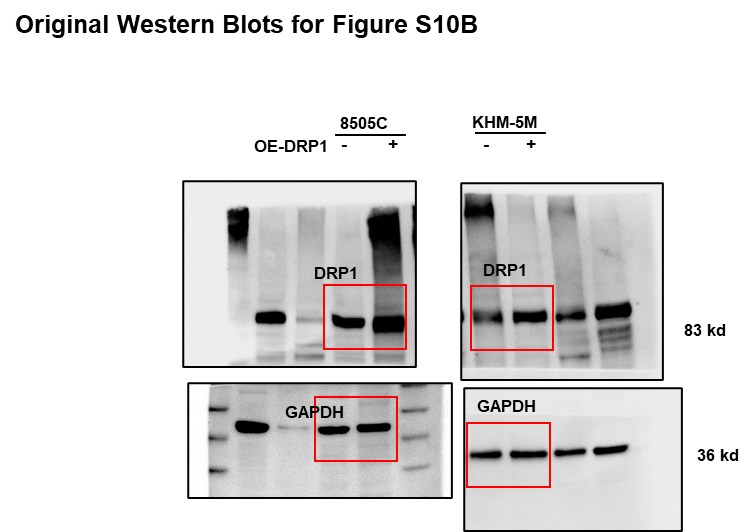

Supplement: Supplementary file 4 — Original Data File [file 41419_2024_6511_MOESM4_ESM.docx]
